# Genetic introgression between different groups reveals the differential process of Asian cultivated rice

**DOI:** 10.1038/s41598-022-22674-3

**Published:** 2022-10-21

**Authors:** Hao Gong, Bin Han

**Affiliations:** 1 School of Life Science, Huizhou University, Huizhou, 516007 China; 2 National Center for Gene Research, State Key Laboratory of Plant Molecular Genetics, Center for Excellence in Molecular Plant Sciences, Institute of Plant Physiology and Ecology, Chinese Academy of Sciences, Shanghai, 200233 China

**Keywords:** Agricultural genetics, Genetic variation

## Abstract

The Asian cultivated rice consists of two major subspecies: *indica* and *japonica*. There are already many reports about the existence of genetic introgression between the two subspecies. They propose some possible introgression-related genes from the comparison of population parameters. This study uses the genome-wide variation data of Asia cultivated rice to investigate their genetic introgression on the whole genome level. We detect a total of 13 significantly high introgression loci between the *tropical japonica* and *indica* populations. Two different methods are used to identify the genetic introgression regions. For most of the detected introgression regions, they generally get consistent results. Some previously known introgression genes are detected in the identified introgression loci, such as heat resistance gene *TT1* and *GLW7*. The biological functions for these genetic introgression regions are annotated by the published QTL mapping results. We find that genetic introgression plays a vital role in the determination of both the phenotype and the domestication process of different groups. Our study also provides useful information and resources for the study of rice gene function and the domestication process.

## Introduction

Genetic introgression is the spread of alleles from one species into the gene pool of another through backcrossing with members of parental lines. Many generations of hybridization and back-crossing are needed to make the introgression alleles stable^1–5^. It can be divided into the human-mediated process and the natural process. The breeding process of introducing the segments that control favorable traits from one population to another is an evident process of human-mediated introgression^6^. Human-mediated introgression is prevalent in the animal and crop domestication process. Natural introgression often happens in adapting to the local environment^7–15^. For example, recent reports show that the early Tibetans acquire the allele to improve the binding of hemoglobin to the oxygen from the Denisovan like ancient human population^8^. There are also reports of genetic introgression in the cultivated maize from the wild maize in the highland of Mexico. They discover widespread wild maize genome components in the cultivated maize genome^16^. By transmitting the introgression segment of wild maize to the cultivated maize, they show that these segments can improve the plant height and pigment extension. In this study, we try to characterize the genetic introgression within the subgroups of the cultivated rice using the whole genome variation data of cultivated rice.

Many previous studies have shown that the domestication of Asian cultivated rice is of single-origin^17,18^, which proposed the cycles of introgression hypothesis. Based on this theory the wild rice was first domesticated to ancient *japonica*. One part of ancient *japonica* hybridizes with the local wild rice in south Asia to form the *indica* subgroup. Another ancient *japonica* group is domesticated to the modern *japonica* group. The *japonica* subgroup has a wide distribution in East Asia. It’s divided into the *temperate japonica* and *tropical japonica* subgroups. Here we analyze the genetic introgression within the Asian cultivated rice groups. However, our study mainly focuses on the genetic introgression between the *tropical japonica* and *indica* population. Though the *tropical japonica* clusters with the *temperate japonica* in the whole-genome phylogenetic tree analysis, this subgroup is similar to the *indica* subgroup on some phenotypes. For example, heat resistance, grain shape, and plant height.

Two different methods are used to detect the genetic introgression within the cultivated rice. The first one is the phylogenetic tree method^19,20^. The second one is the D-static method^1,2^. Different from the phylogenetic tree method, the D-static method can calibrate the disturbance of the same domestication origin of cultivated rice in the detection of introgression segments by using the genetic variants in the ancient outgroup as the background. The two detection methods get similar results in the introgression regions within the cultivated rice.

To analyze the biological function of these introgression segments in cultivated rice, we use QTL mapping results of a hybrid population constructed with *indica* and *japonica* to annotate these regions. Here we use the genome component matrix of the cultivated rice population to conduct admixture mapping to detect the phenotype-related introgression regions^21^. Many previously reported phenotype-related genes are located in the introgression region. Our study systemically analyzes the genetic introgression within the cultivated rice. By using results of admixture mapping and QTL mapping, the biological functions for these introgression regions are also investigated.

## Results

### Primary study of genetic introgression within the cultivated rice with phylogenetic tree method

**Figure 1:**
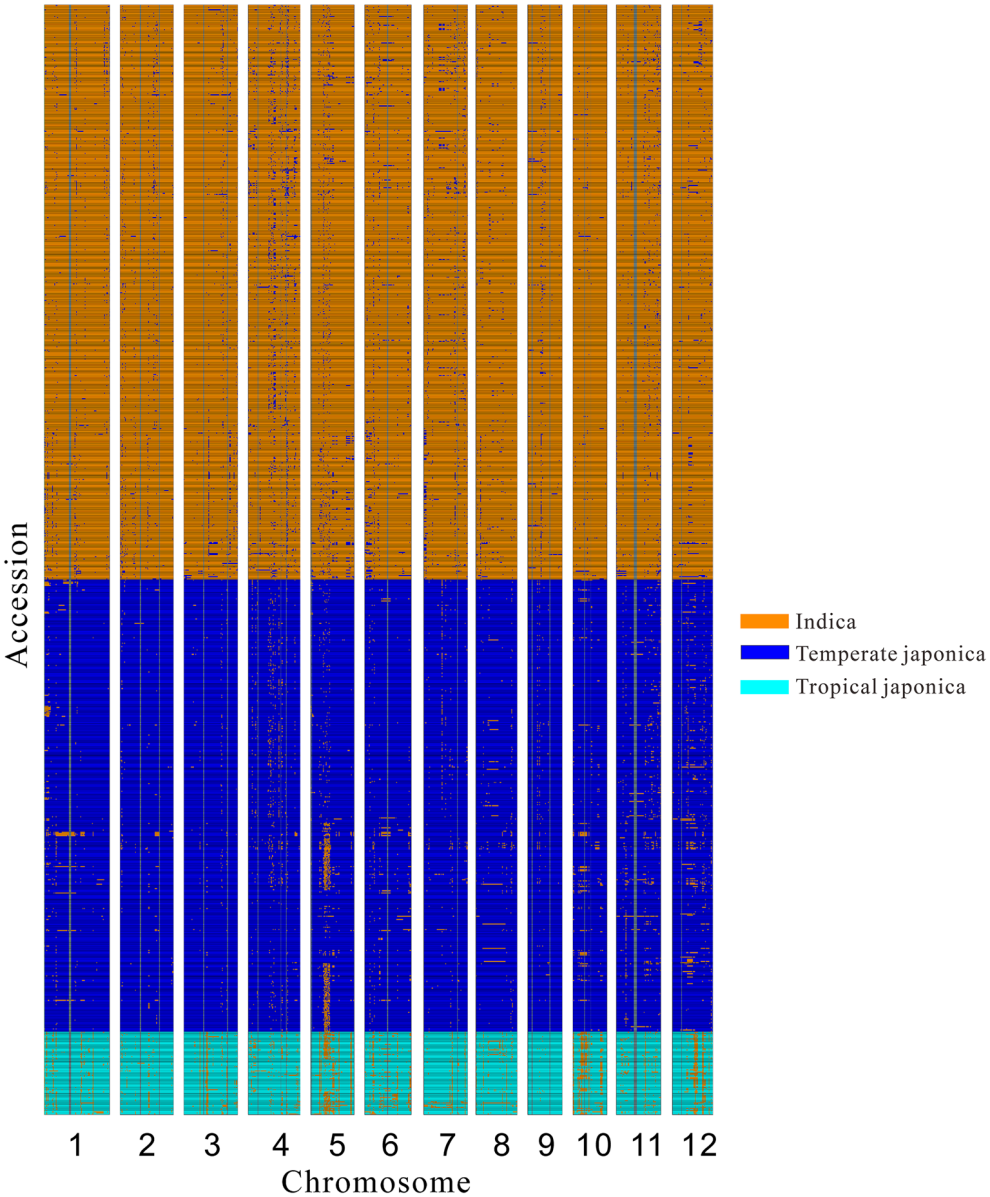
Diagram for the genetic introgression in the major three subgroups of Asia cultivate rice. The horizontal line represents 12 chromosomes of the rice genome. The vertical line represents accessions in three groups. Each small horizontal line represents one accession in the cultivated rice population. The small rectangle on the map represents a small block for one accession. Different colors represent different genome components, the gray area represents genetic areas with insufficient polymorphic sites to construct the phylogenetic tree.

By summarizing the introgression accessions across the whole genome with the phylogenetic tree method, we construct the map of genetic introgression for the different groups. The introgression regions occupy only a small part of the entire genome (3%). However, some blocks in the genome are enriched with genetic introgression. There is genetic introgression in nearly all accession in a few blocks (Fig. 1). As it’s difficult to get high genotyping accuracy in the high repeat sequence of the genome, it may disturb the construction of neighbor-joining tree and the genetic introgression detection process. By comparing the genetic introgression level in the high repeat regions with that of the whole genome level (Supplementary Table 3), we find no significant enrichment of introgression in the high repeat sequence of the rice genome (Chisq.test, *P* value 0.1712).

**Figure 2:**
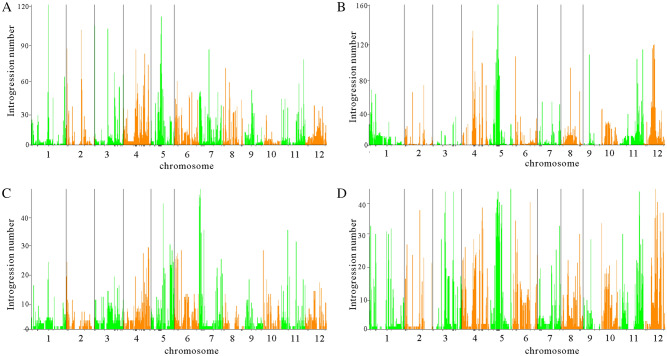
Summary of the cultivated rice accessions that exist introgression between different groups. The horizontal line for each diagram represents 12 chromosomes of the whole rice genome. The vertical line represents the number of accessions with genetic introgression between different groups. The black line under the horizontal line represents the 55 major selective sweeps detected by the previous study. (**A**) represents summary data for genetic introgression from *temperate japonica* to *indica*. (**B**) represents the summary data for the genetic introgression from *indica* to *temperate japonica*. (**C**) represents the summary data for the genetic introgression from *tropical japonica* to the *indica*. (**D**) represents the summary data for the genetic introgression from *indica* to *tropical japonica*.

**Figure 3:**
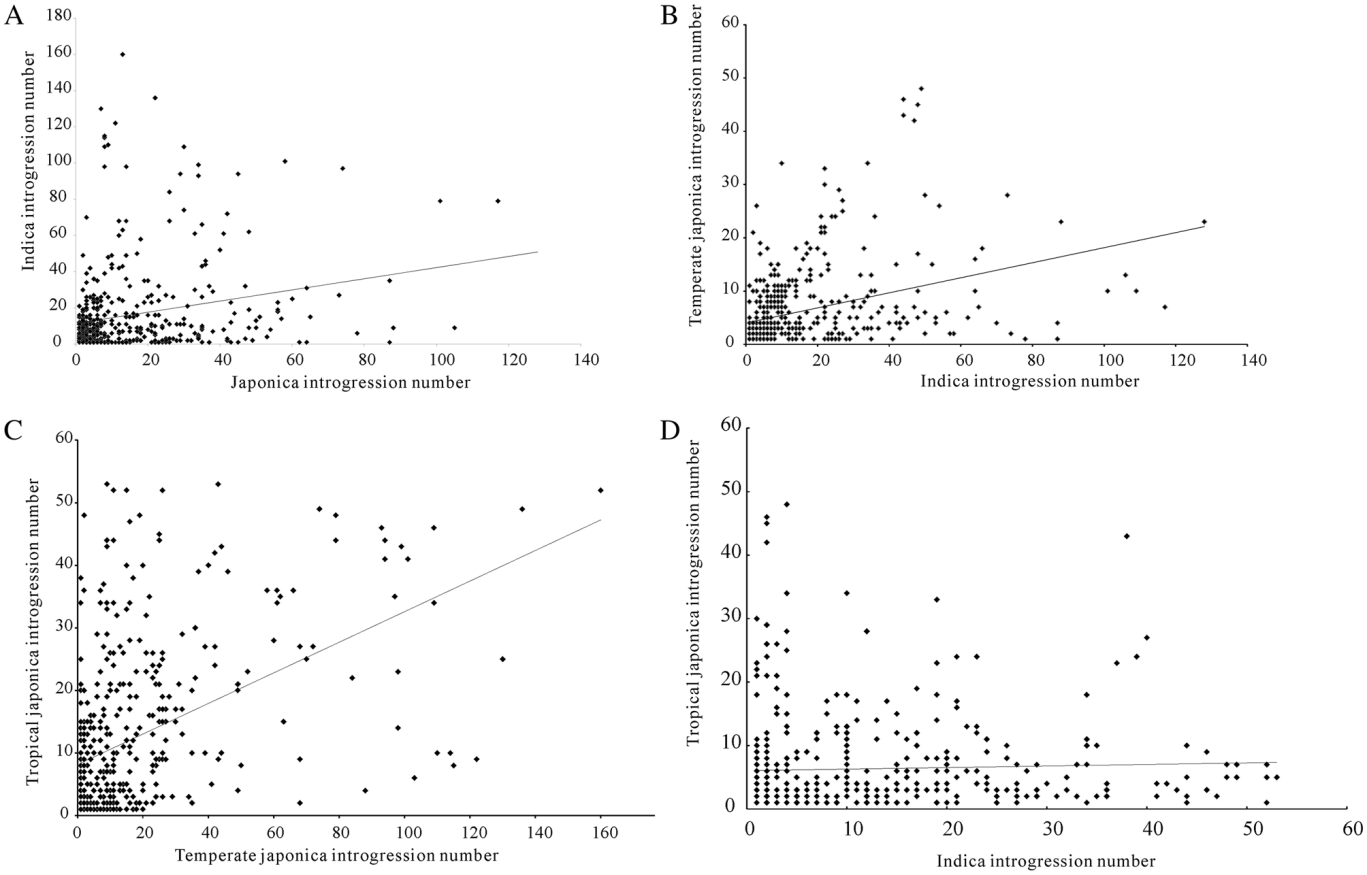
Correlation of introgression segments between different subgroups in all blocks. The horizontal line and vertical line represent different introgression number for different groups. The black line on the figure represents the regression line of the introgression data. The groups they represent on each line are labeled in the figure.

To get a clear view of the genetic introgression level within the cultivated rice, the introgression accession number on each block is summarized. We find that some regions exist high introgression levels from the *indica* subgroup to the *tropical japonica* subgroup (Supplementary Fig. 1). However, we detect few introgression accessions from the *tropical japonica* subgroup to the *indica* subgroup in the same introgression regions (Supplementary Table 2). As the introgression level within different subgroups of cultivated rice (Fig. 2) had been characterized, we also check the correlation of the introgression segments between the four groups at the whole genome level. Interestingly, no significant correlation of introgression segments is found between the two different groups (Fig. 3). There are no consistent regions for the high introgression regions in different groups except one in the centromere of chromosome 5. So the direction of introgression within the two groups is unidirectional. Previous studies have detected selective sweep regions between cultivated rice and wild rice. We find an overlap of the selective sweep regions with our detected high introgression regions (Figs. 2, S17). Some high introgression regions that we detect between different rice subgroups, which don’t overlap with the selective sweep region, may control other important traits that are not under high selection pressure. Besides the overlap of the introgression with the selective sweep regions, we find great variation in the genetic introgression between different groups. The *tropical japonica* and *temperate japonica* are both domesticated from ancient *japonica*. The two subgroups have high genetic distance with the *indica* group and cluster together in the whole genome phylogenetic tree analysis. However, we find significantly more introgression in the direction from *indica* to the *tropical japonica* than that from *indica* to the *temperate japonica* in the genetic introgression analysis (chi-square test, *P* value < 0.01).

Using the permutation test results as the background, we detect 13 regions that contain significantly high genetic introgression in the whole genome level from *indica* to *tropical japonica*. Some previously reported introgression genes are located in our detected high introgression loci. For example, *TT1 GS3*, *GLW7*^22–24^. *TT1* is a heat-resistant gene introduced to the *tropical japonica* from the *indica* by genetic introgression (Fig. S1)^23,25^. Nearly all the tropical accessions (96.2%) culster together with *indica* accessions other than *temperate japonica* accessions in the phylogenetic tree constructed with the variants in *TT1* located blocks. Only a few *indica* accessions (5%) cluster together with the *tropical japonica*. *GLW7*, a grain size-related gene, is found to exist great *F*_*st*_ between the *temperate japonica* and *indica*. However, significantly lower *F*_*st*_ between the *tropical japonica* and *indica* was observed in the *GLW7* gene region. By checking the 1 Mb sequence flanking both sides of the *GLW7* region, we find that the *tropical japonica* accessions that exist introgression have identical haplotype with *indica* accessions (Fig. S2). The phylogenetic tree constructed with the genetic variants in the flanking regions shows that the divergence patterns of different groups confer well with the haplotype map distribution. So our phylogenetic tree method detects the genetic introgression within the rice groups with good accuracy.

### Characterization of the introgression within the cultivated rice with the calibration of the wild rice

**Figure 4:**
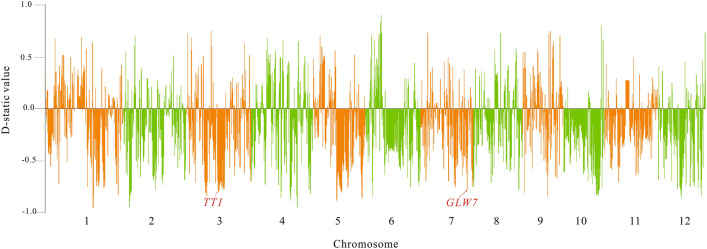
Genetic introgression between the *indica* and *tropical japonica* population detected by the D-static value method. The horizontal line represents 12 chromosomes of the rice genome. The vertical line is the D-static value of the two groups. *TT1* and *GLW7* are two known genes that exist genetic introgression between the *tropical japonica* and *indica* population.

There may be an existence of analysis bias to detect the genetic introgression regions with only the phylogenetic tree method, so we also apply another different D-static method to characterize genetic introgression within different groups to make a comparison. We have found more genetic introgression between *tropical japonica* and *indica* with the phylogenetic tree method, which may be caused by the overlap of their distribution areas (Southside of East Asia). Previous reports have found that the domestication of Asia cultivated rice population is single-origin. Some genome segments are conserved within different cultivated rice groups. As the conserved segments may disturb the phylogenetic tree analysis, D-static is a useful way to calibrate these effects by using the outgroup species as the background. As our study has found extensive introgression between *tropical japonica* and *indica*, we decide to focus on the introgression between the two groups (Fig. 4). The detailed settings for the four groups used by the D-static calculation are illustrated in the methods^25^. We have also tried to use the wild rice as outgroup to calculate the D-static value for the cultivated rice group. However, it results in too many introgression regions across the genome. We think that the extensive introgression detected by using the wild rice as the outgroup may be caused by the conserved genome segments of wild rice in the cultivated rice. The African rice is reported to have great genetic distance with the Asian cultivated rice. Only few genetic introgression regions are detected between the African cultivated rice and Asian cultivated rice in the previous study. So we set the African cultivated rice as outgroup in the D-static calculation.

After calculating the D-static value between the *tropical japonica* and *indica* population, we also find enrichment of genetic introgression in some regions of the rice genome. The large absolute D-static value means higher genetic introgression in the studied population compared with the reference population. Some high introgression blocks detected in the phylogenetic tree method overlap with the introgression peaks detected in the D-static method (Supplementary Table 4). There is also excessive-high genetic introgression in some known introgression gene regions. The highest introgression peak in chromosome 3 contains the known heat resistance gene *TT1*, the genetic introgression peak of chromosome 7 contains the known gene *GLW7* (Fig. 4). For the high genetic introgression regions from *indica* to the *tropical japonica*, nearly no genetic introgression is detected in the direction from *tropical japonica* to the *indica*. So it again proves that the detected genetic introgression is unidirectional, which is consistent with the results detected using the phylogenetic method. Unlike the phylogenetic method, the genetic introgression region detected in the D-static method is wider than those detected in the phylogenetic method. As similar broad genetic introgression regions are also detected in the other reports using the D-static method, this broad introgression region may be caused by the algorithm defining the introgression region. In total, the two methods get consistent results in the detection of genetic introgression regions.

### Common introgression regions detected within the different cultivated rice subgroups

**Figure 5:**
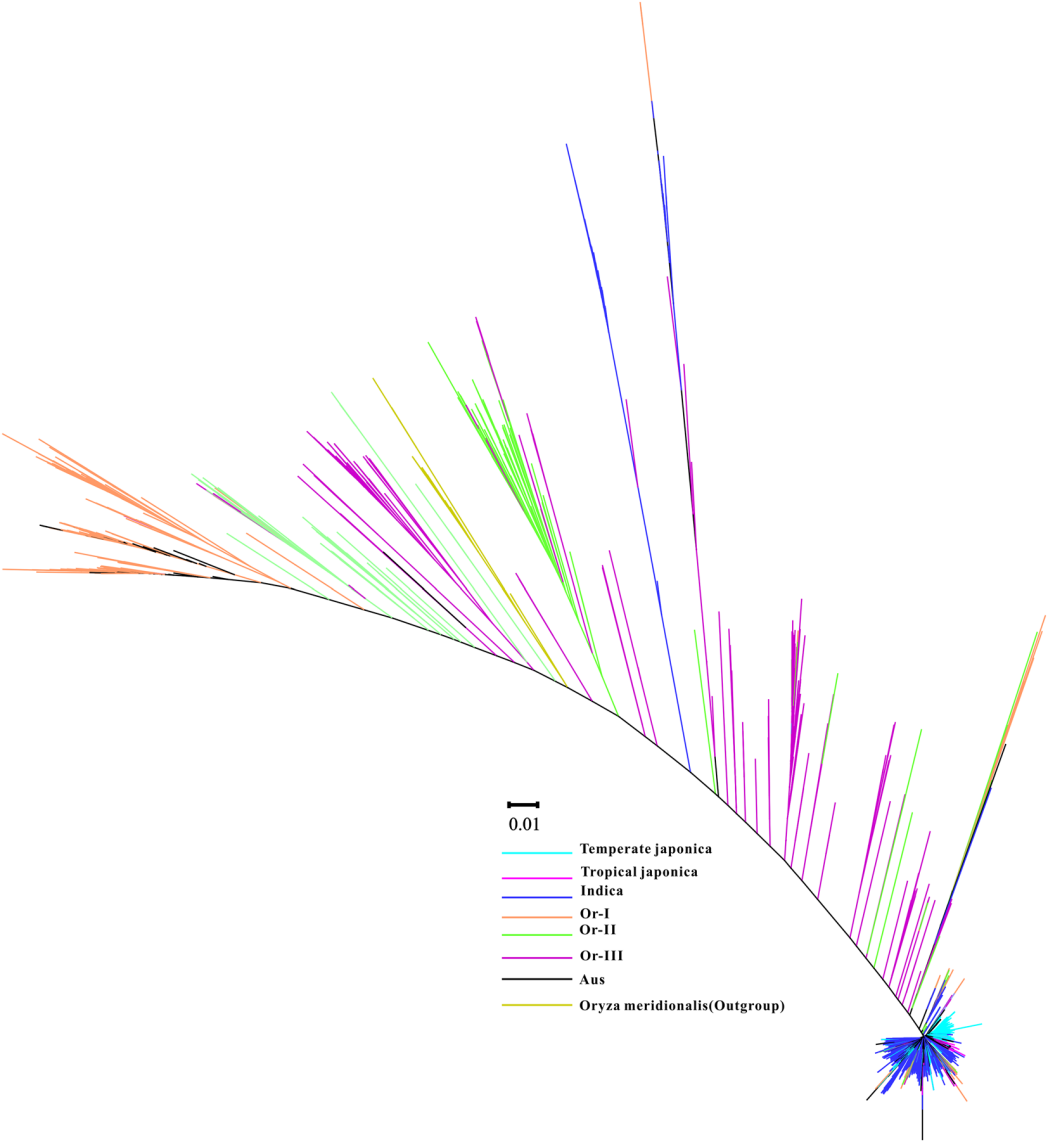
The neighbor-joining tree constructed with the genetic variants located in the centromere of chromosome 5. The genetic variants located in the region from 9.5 to 13.5 Mb are used to build the phylogenetic tree. Each subgroup of the wild rice is labeled with a different color.

After analyzing genetic introgression regions within different rice subgroups, we find 13 consistently high genetic introgression in the rice rice genome, where a 4 Mb region (9.5–13.5 Mb) near the centromere of chromosome 5 was the largest one (Supplementary Tables 1, 5–6). It’s found to be a high sequence repeat region from the annotation of the Nipponbare reference genome^26^. The previous report has found this region under strong selective pressure^18^. We want to know whether the consensus high introgression region is the conserved segment of the ancient wild rice species or introgression from other cultivated rice populations. Constructing phylogenetic trees with the cultivated rice and related wild rice progenitor was used to separate these two kinds of introgression events. Based on the cycles of introgression model the *japonica* population is found to be domesticated from the Or-III wild rice group. The *indica* population is domesticated from the Or-I wild rice group. The genetic introgression between the Or-I wild rice group and the *indica* population as well as those between the Or-III group and *japonica* population are also analyzed. By comparing the introgression events detected between the progenitor, their relative the ancient introgression and introgression after separation would be separated (Supplementary Fig. 18). High genetic introgression is found between the Or-I wild rice group and the *indica* population. However, few genetic introgression accessions are detected between the *japonica* population and the Or-III wild rice group. So this introgression may happen in the recent intrgoression. To further dissect the evolutionary relationship of this consensus introgression region, we construct a phylogenetic tree with the genetic variants located in the region from 9.5 to 13.5 Mb using the *Oryza meridionallis* as the outgroup (Fig. 5). The phylogenetic tree shows that all the cultivated rice accessions cluster together in a small region, consistent with the previous result of low genetic diversity in the cultivated rice. Though the cultivated rice is generally divided into the *indica* subgroup and *japonica* subgroup, we find a mixture of some rice accessions between the two groups in the constructed phylogenetic tree. The Or-III wild rice subgroup has the closest relationship to the cultivated rice among all the wild rice populations. Interestingly, Or-I wild rice subgroup was found to have the greatest distance to cultivated rice accessions. However, these results are different from the phylogenetic tree constructed with the whole genome variation data, which shows the divergence of *indica* and *japonica* subgroups. The Or-I and Or-III wild rice subgroup cluster together with the two different cultivated rice subgroups. It may suggest a closer relationship for Or-III with the *indica* and *japonica* population in this consistent introgression region.

Besides the phylogenetic tree, genetic diversity between different groups can also be used to dissect the relationship of different populations. We decide to study the genetic diversity in different rice groups to track the origin of this region. As we find different genetic diversity in the three rice groups (*indica*, *japonica,* and wild rice), we decide to characterize the whole genome genetic diversity in small groups of our collected materials to make a detailed comparison. All the small groups of wild rice have high genetic diversity in the region from 9.5 to 13.5 Mb. In contrast, all the cultivated rice small groups have low genetic diversity in this region except the *aus* cultivated group. *aus* is found to originate from the cross of the Or-I wild rice group and Ancient *japonica* in the previous report, which is similar to the *indica* population. So the origin of the consensus introgression in the cultivated rice may be from the Or-III group. It’s under high human selection pressure, which is conserved in all the cultivated rice population in the rice domestication process. However, in the formation of *indica* and *aus* population, there is introgression of some genome segments from the Or-I group to the *indica* population and *aus* population. As most *aus* accessions cluster with the Or-I group other than *indica* and *japonica* population in the phylogenetic tree analysis (Fig. 5), it suggests more genetic introgression from the Or-I wild rice group in the *aus* than that of the *indica* population in this region.

### Characterization of the biological functions for the introgression region using admixture mapping method

Admixture association mapping studies had been reported in many species, such as human, dog, maize, etc. It’s often used to locate the loci that cause special phenotypes between different groups. The African American is an excellent human population for genetic association mapping between the black man population and the white man population^27^. Many admixture mapping association studies had been conducted in this population to study the cardiovascular, body mass index, and other diseases to locate disease-related alleles.

**Figure 6:**
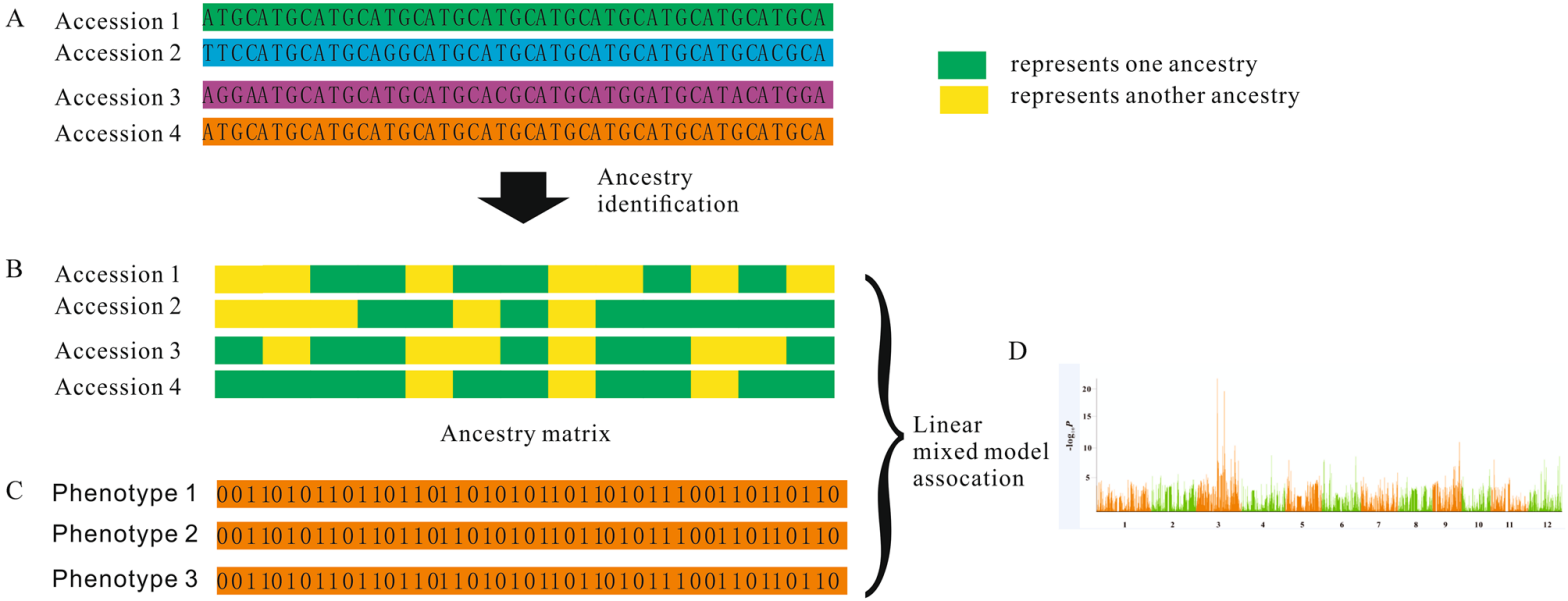
Workflow for the admixture association mapping in the cultivated rice. (**A**) shows the haplotype map of four rice accessions. (**B**) shows the genome component detected by the Admixture software. The green color and yellow color represent the two different genome components. (**C**) represents the phenotype data for different accessions. (**D**) shows the Admixture mapping results by associating the Ancestry component matrix with the phenotype.

Unlike the whole genome association study, which associates the genotype with the phenotype, the genome components of different populations are associated with the phenotype to locate the phenotype-related region in the admixture association mapping analysis. If there is no genetic introgression in the region associated with this phenotype, we can’t locate the phenotype-related region by admixture association mapping in a population even with large phenotype differences between the two subgroups. As there are few reports about admixture mapping association analysis in the crops, our study decides to characterize genetic admixture within the cultivated rice population. As our study has found some excessive genetic introgression loci between the *indica* and *japonica* population, we choose to focus on the admixture association mapping between the *indica* and the two *japonica* subgroups (*temperate japonica* and *tropical japonica*). We conduct admixture association mapping with 11 previously published Asia cultivated rice agronomic traits (Figs. 6, S4–S14). Many phenotype-related regions detected in the admixture association mapping are also reported in the previous genome-wide association studies using the same phenotype. Some known phenotype-related genes are located in the peak association regions of admixture mapping. For example, there are *GS3*, *AlK*, *Waxy,* and others^23,24,28–32^. So we can find that many phenotype-associated regions detected in GWAS exist genetic introgression in the small blocks between *indica* and *japonica* (Figs. S15–16). In this study, we take the amylose content and grain length as an example to explain the results of admixture mapping.

**Figure 7:**
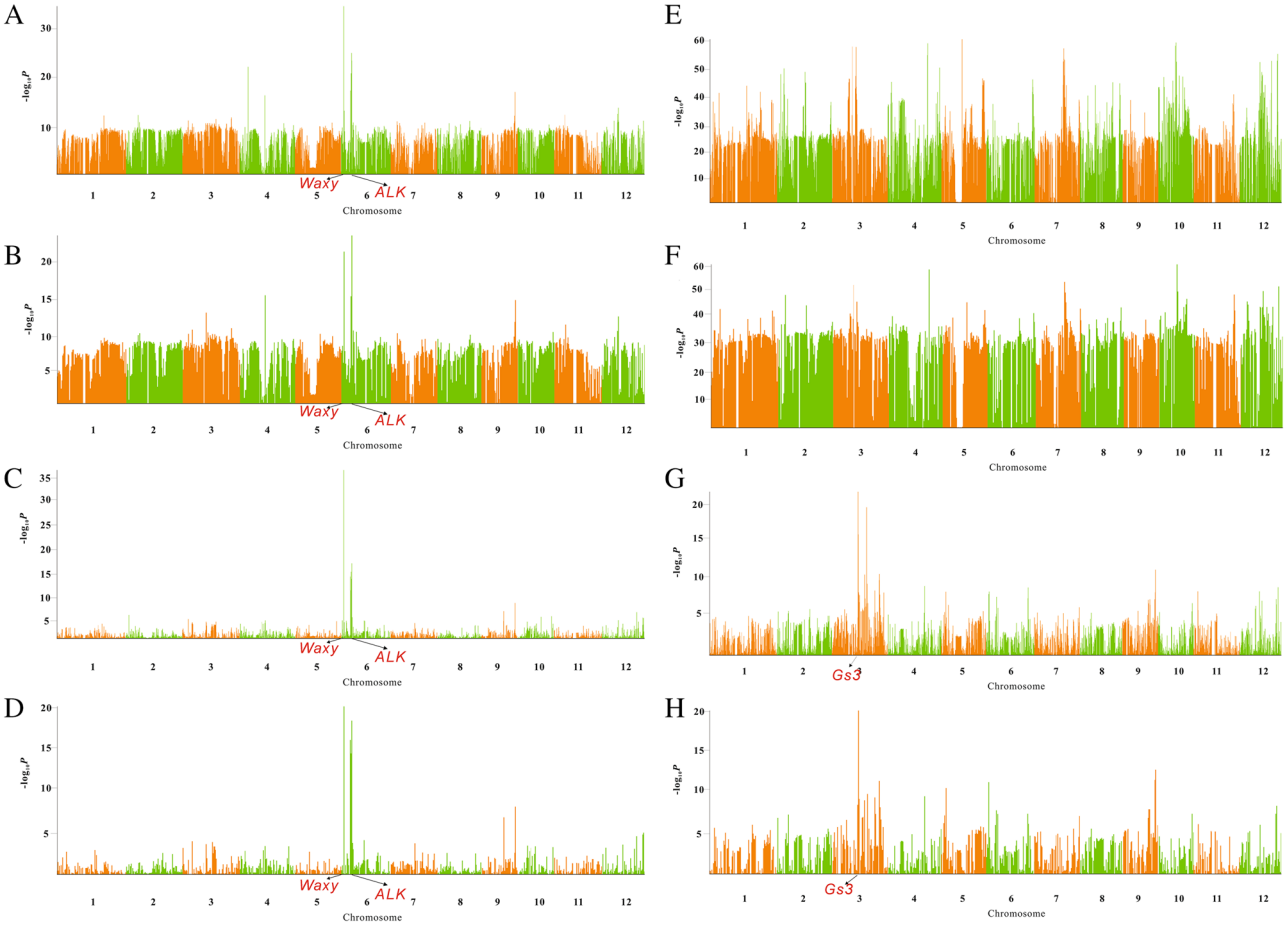
Admixture association mapping results using amylose content phenotype and the grain length phenotype. (**A**–**D**) show the admixture association mapping results for the amylose content phenotype. (**E**–**H**) show the association results for the grain length phenotype. (**A**) shows the association result of the *indica* component detected in the merged population of *indica* and temperate population. (**E**) shows the result of grain length phenotype with the same data. (**B**) shows the association result of the *temperate japonica* component detected in the merged population of *temperate japonica* and *indica*. (**F**) shows the result of grain length phenotype with the same data. (**C**) shows the association result of the *tropical japonica* component detected in the merged population of *tropical japonica* and *indica*. (**G**) shows the association of grain length phenotype using the same data. (**D**) shows the association result of the *indica* component detected in the merged population of *tropical japonica* and *indica*. (**H**) shows the association result of grain length using the same data.

There are two evident association peaks in the admixture mapping association results between the *indica* and *tropical japonica* population for the amylase content phenotype. There are also two association peaks in the same region in previous genome association study with the same trait. Two known phenotype-related genes, *Waxy* and *ALK*, are located around the peaks (Fig. 7). The two association peaks can only be detected by GWAS in the *indica* population and whole population. However, they can’t be detected by GWAS in the *japonica* population for the low genetic diversity. Similar results are also found in admixture association mapping results. Strong sharp peaks are detected in the association results between the *indica* and *tropical japonica* populations. However, moderate peaks are detected in the association results between the *indica* and *temperate japonica* population. There are two different alleles for the *Waxy* gene in the rice genome, and they are *Wx*^*a*^ and *Wx*^*b*^. The *Wx*^*a*^ type is an ancient type that exists in the wild rice population and indica population. *Wx*^*b*^ is a variant type that exists in the japonica population. So our admixture mapping results show that there is genetic introgression between the *indica* and tropical japonica populations in our collected materials. These genetic introgression regions between different groups are also validated by our phylogenetic tree method.

Similar results are found in the grain length phenotype admixture association mapping results. There are no evident association peaks in the association results between the *indica* and *temperate japonica* population. However, strong association peaks are found between the *indica* and *tropical japonica* populations. The known grain length-related gene *GS3* is located in the association peaks of chromosome 3 (Fig. 7). *GS3* was found to exist genetic introgression between the *indica* and tropical population in the previous study (Fig. S3). The phenotype-related regions detected by our admixture association mapping method are highly reliable introgression loci between different groups. Genetic introgression may introduce many phenotype-related loci of rice subgroups from other rice populations. Besides the overlapping of association peaks with the GWAS results, our study also detects many phenotype-related regions that are missed by the GWAS method. These new phenotype-related candidate regions can be further investigated by constructing a related hybrid population to further study their biological functions.

## Discussion

Genetic introgression is often used in the human population to explore the genetic flow between populations. It can be used to locate the alleles responsible for the population-specific phenotypes^2,27^. There are also reports in crops, such as maize. They detect introgression between cultivated maize and wild maize^16^. Our study characterizes genetic introgression within cultivated rice subgroups at the whole genome level with high-density SNP markers. We get consistent results of genetic introgression regions between different cultivated rice groups with two different methods. Excessive genetic introgression is found between the *indica* population and *tropical japonica* population, which may be caused by the overlap of distribution areas. They both have distribution in the south side of East Asia. So the *tropical japonica* may originate from ancient *japonica* by genetic introgression and forms a special ecotype after acquiring many alleles from the *indica* to adapt to the local environment in the south side of East Asia. For example, *TT1,* which enhances the heat resistance ability of *tropical japonica*, is located in the highest genetic introgression peaks between *indica* and *tropical japonica*^23^. By analyzing the flanking sequence around the *TT1* locus, we find that the *tropical japonica* has identical haplotypes with the *indica* population. Using the admixture association mapping method, we find that some agronomic traits-related regions also have a moderate level of genetic introgression. They are also validated by some known introgression genes (*Waxy*, *ALK*, and *GS3*). Some admixture association peaks can only be detected between the *indica* and *tropical japonica* populations. It may suggest that the *tropical japonica* population gets some phenotype-related loci from the *indica* by genetic introgression.

The genetic region near the centromere of chromosome 5 was found to show high genetic introgression in different groups. By phylogenetic tree analysis, we find that this region may originate from the Or-III wild rice group in the cultivated rice for its closest relationship with the cultivated rice (Fig. 5). After analyzing the genetic introgression between the cultivated rice and their related progenitors, we detect no genetic introgression between Or-III and the *japonica*. Only moderate genetic introgression is found between the Or-I and *indica*. By analyzing the genetic diversity within the different groups of rice accessions, we find low genetic diversity in the whole cultivated rice except the *aus* population. Most of the *aus* accessions cluster with the Or-I group in the phylogenetic tree constructed with the genetic variants located in the consensus high introgression region. So this region may originate from the Or-III group in the cultivated rice. It’s conserved in the cultivated rice population after domestication selection. Some genome segments of the *indica* population are introduced to the *tropical japonica* and *aus* population by genetic introgression from the Or-I group in the domestication process. Our study characterizes the genetic introgression within the cultivated rice groups at the whole genome level. These introgression regions are annotated by the admixture association mapping study, which provides a good resource for investigating the gene function and genetic markers for the breeding process.

## Methods

1. Materials and phenotypes
  One part of the sequence data used in this study and whole phenotype data is from the previous studies^18,33^. Their average sequence coverage is about one fold. We download 341 *tropical japonica* accessions and 199 *aus* accession from the rice 3 K genome project^34^. 88 African cultivated rice sequence data is also downloaded from the public database^25^. Sratoolkit converts the binary sra format to the text fastq format.
2. Genotyping from the low coverage sequence data
  Smalt (version 0.75) software is used to align all the short sequence reads to the rice reference genome (IRGSP version 4) to generate the aligned cigar format. Self-customized perl scripts are used to filter the aligned results. Only sequences that have a unique match are conserved. The sequence alignments that have low mapping score (< 60) and low mapping rate (< 82% of the total length) are filtered. We also exclude the sequence with a mismatch rate more prominent than 8% of the full length. Using the id of the filtered sequence, we retrieve the reads sequence from raw sequence data. Pileup software (version 0.4) piles up the filtered reads to call SNP from the sequence data. For the detected single nucleotide polymorphism of the whole population, we only keep the polymorphic sites with minor allele frequencies greater than 5% and the missing rate smaller than 40%.
3. Characterization of genetic introgression using the phylogenetic tree method
  In our study, the whole genome variation data of the cultivated rice is divided into 500 kb non-overlapping blocks. Using the self-customized C+ + program, we generate a kinship matrix for the population in the small block. The kinship matrix is converted to the input format of phylip (version 3.69). The neighbor module of the phylip software is used to generate the phylogenetic tree for each small block. The phylogenetic tree using the whole genome variation data is first used to determine the group for each accession. The ape package of the R language is used to present this phylogenetic tree and label them with different colors according to the subgroup they belong to. For the two groups that we want to characterize genetic introgression, we set them as group A and group B. Some accessions belonging to group A in the whole genome level cluster with group B in the small block. These accessions are found to have genetic introgression from group B to group A in this block.
4. Characterization of genetic introgression with D-static value
  Our study also uses the previously reported “ABBA-BABA” method (D-static method) to characterize the genetic introgression^1,2^. It needs four populations to calculate the D-static value. We label them as {P1,P2,P3,O}. P1 and P3 are the groups that we want to detect genetic introgression. P2 is an ancient population that is similar to P1 and P3. O is the ancient outgroup. We calculate the allele frequency for the four different groups. The following equation is used to calculate the D-static value^1^.
  $${\text{D}}\left( {{\text{P1}},{\text{P2}},{\text{P3}},{\text{O}}} \right) = \frac{{\mathop \sum \nolimits_{{{\text{i}} = 1}}^{{\text{n}}} \left[ {(1 - {\text{p}}_{{{\text{i}}1}} ){\text{p}}_{{{\text{i}}2}} {\text{p}}_{{{\text{i}}3}} \left( {1 - {\text{p}}_{{{\text{i}}4}} } \right) - {\text{p}}_{{{\text{i}}1}} \left( {1 - {\text{p}}_{{{\text{i}}2}} } \right){\text{p}}_{{{\text{i}}3}} \left( {1 - {\text{p}}_{{{\text{i}}4}} } \right)} \right]}}{{\mathop \sum \nolimits_{{{\text{i}} = 1}}^{{\text{n}}} \left[ {(1 - {\text{p}}_{{{\text{i}}1}} ){\text{p}}_{{{\text{i}}2}} {\text{p}}_{{{\text{i}}3}} \left( {1 - {\text{p}}_{{{\text{i}}4}} } \right) + {\text{p}}_{{{\text{i}}1}} \left( {1 - {\text{p}}_{{{\text{i}}2}} } \right){\text{p}}_{{{\text{i}}3}} \left( {1 - {\text{p}}_{{{\text{i}}4}} } \right)} \right]}}$$
  In our study, we set *tropical japonica* as P1, *temperate japonica* as P2, *indica* as P3, *Oryza glaberrima* (African cultivated rice, P4) as the O. The equation above is used to calculate the D-static value. ${\mathrm{p}}_{\mathrm{i}1}$, ${\mathrm{p}}_{\mathrm{i}2}$, ${\mathrm{p}}_{\mathrm{i}3}$, and ${\mathrm{p}}_{\mathrm{i}4}$ are the observed allele frequency of SNP i in the related population.
5. Admixture association mapping with 11 agronomic traits
  The whole-genome variation data is sliced into 200 kb non-overlapping blocks. The genetic variants are converted to the ped and map format for Admixture^21^ using self-customized perl scripts. The genome component for the different groups is determined for all the accessions. The genome components matrix of the whole population is used to associate with phenotype using a linear mixture model to generate the final association results.

## Supplementary Information

Supplementary Information.

## Data Availability

The datasets supporting the conclusions of this paper are included within the article and its additional files. The cultivated rice sequence data were downloaded from the EBI European Nucleotide Archive with ERP000729 and ERP000106. The data for the wild rice accession was downloaded from the EBI with ERP001143, ERP000729, and ERP000106.
