# Supplementary material for: Genetic introgression between different groups reveals the differential process of Asian cultivated rice
Source: Sci Rep. 2022 Oct 21;12:17662. doi: 10.1038/s41598-022-22674-3 (PMC9587041; doi:10.1038/s41598-022-22674-3)
Supplement: Supplementary file 1 — Supplementary Information. [file 41598_2022_22674_MOESM1_ESM.docx]

**Supplementary tables and figures**

**Table of contents**

**Supplementary Table 1**. Genetic introgression accession between different groups in the consistent high genetic introgression region.

**Supplementary Table 2**. The table for the 13 high introgression sites from *indica* to *tropical japonica* detected in this study.

**Supplementary Table 3**. The distribution of introgression accession number between the high repeat region and whole genome

**Supplementary Table 4**. Table for high genetic introgression regions detected by the D-static method compared with the phylogenetic tree method.

**Supplementary Table 5** Table for the genome coordinate of the 13 consistently high genetic introgression regions between the three subgroups of rice population

**Supplementary Table 6** Table for the introgression ratio within the rice subgroups and their progenitors in 13 consistently high genetic introgression regions

**Supplementary Fig. 1**. Neighbor-joining tree constructed with the 500kb flanking genetic variants around the *TT1* locus.

**Supplementary Fig. 2**. Diagram for the haplotype around the 1Mb flanking genetic variant around the GLW7 locus.

**Supplementary Fig. 3**. Genetic introgression in the GS3 locus between the *indica* subgroup and the japonica group.

**Supplementary Fig. 4**. Admixture association mapping results using heading date phenotype.

**Supplementary Fig. 5**. Admixture association mapping results using amylose content phenotype.

**Supplementary Fig. 6**. Admixture association mapping results using pericarp color phenotype.

**Supplementary Fig. 7**. Admixture association mapping results using tip color phenotype.

**Supplementary Fig. 8**. Admixture association mapping results using glue color phenotype.

**Supplementary Fig. 9**. Admixture association mapping results using protein content phenotype.

**Supplementary Fig. 10**. Admixture association mapping results using gelatinization temperature phenotype.

**Supplementary Fig. 11**. Admixture association mapping results using one thousand grain weight(TGW) phenotype.

**Supplementary Fig. 12**. Admixture association mapping results using grain number per panicle phenotype.

**Supplementary Fig. 13**. Admixture association mapping results using grain length phenotype.

**Supplementary Fig. 14**. Admixture association mapping results using grain width phenotype.

**Supplementary Fig. 15**. The introgression between the *indica* and the *tropical japonica* overlaps with the rice known agronomically important genes.

**Supplementary Fig. 16**. The introgression between the *indica* and the temperature japonica overlapping with the rice known agronomically important genes.

**Supplementary Fig. 17**. Summary of the cultivated rice accessions that exist introgression between different groups in circles.

**Supplementary Figure18.** Summary of the genetic introgression between the rice subgroup and their wild rice progenitors.

| **Supplementary Table 1**. Genetic introgression accession between different groups in the consistent high genetic introgression region | | | | | | | | | | | | | | |  |
| --- | --- | --- | --- | --- | --- | --- | --- | --- | --- | --- | --- | --- | --- | --- | --- |
| Group | Chr5,9.5-10.0Mb | | Chr5,10.0-10.5Mb | | Chr5,10.5-11.0Mb | Chr5,11.0-11.5Mb | | Chr5,11.5-12.0Mb | | Chr12.0-12.5Mb | Chr12.5-13.0Mb | | Chr13.0-13.5Mb | |  |
| Temp in ind **Vs** jap | 117 | 118 | | 103 | | 123 | 62 | | 76 | | | 82 | | 113 | |
| Trop in ind **Vs** jap | 41 | 46 | | 43 | | 47 | 33 | | 25 | | | 30 | | 44 | |
| Ind in ind **Vs** jap | 28 | 14 | | 30 | | 25 | 49 | | 41 | | | 46 | | 74 | |
| Temp in trop **Vs** Or-III | 2 | 1 | | 1 | | 2 | 6 | | 3 | | | 2 | | 2 | |
| Or-III in temp **Vs** Or-III | 9 | 14 | | 15 | | 16 | 20 | | 25 | | | 17 | | 12 | |
| Trop in trop **Vs** Or-III | 0 | 0 | | 3 | | 0 | 4 | | 3 | | | 2 | | 0 | |
| Or-III in trop **Vs** Or-III | 8 | 14 | | 12 | | 16 | 18 | | 11 | | | 12 | | 11 | |
| *Indica* in ind **Vs** Or-I | 82 | 39 | | 67 | | 40 | 52 | | 60 | | | 52 | | 28 | |
| Or-I in *indica* **Vs**Or-I | 9 | 33 | | 10 | | 34 | 27 | | 19 | | | 28 | | 34 | |

**Supplementary Table 2**. The table for the 13 high introgression sites from *indica* to *tropical japonica* detected in this study.

| chromosome | block id | start | end | tropical japonica introgression | Indica introgression |
| --- | --- | --- | --- | --- | --- |
| 1 | 68 | 33500013 | 33999980 | 72 |  |
| 3 | 23 | 11000024 | 11499949 | 75 |  |
| 3 | 32 | 15500099 | 15999986 | 72 | 1 |
| 3 | 33 | 16000041 | 16499924 | 69 | 1 |
| 5 | 13 | 6000060 | 6499986 | 75 | 15 |
| 5 | 40 | 19500010 | 19999946 | 75 | 1 |
| 5 | 57 | 28000036 | 28499964 | 73 |  |
| 6 | 46 | 22500029 | 22999982 | 56 | 25 |
| 7 | 58 | 28500040 | 28999964 | 65 | 77 |
| 7 | 40 | 19500036 | 19999999 | 62 | 7 |
| 11 | 41 | 20000047 | 20499919 | 66 | 27 |
| 12 | 32 | 15500049 | 15999987 | 74 | 12 |
| 12 | 43 | 21000035 | 21499990 | 70 | 5 |

**Supplementary Table 3**. The distribution of introgression accession number between the high repeat region and whole genome

| Name | High repeat region | whole genome |
| --- | --- | --- |
| Introgression segment | 3,401 | 29,155 |
| Not introgression number | 103,279 | 908,105 |
| introgression ratio | 3.19% | 3.11% |

**Supplementary Table 4**. Table for high genetic introgression regions detected by the D-static method compared with the phylogenetic tree method.

| Serial | Chro | Start | End | | D-static value | Length | Phylo_start | Phylo_end | Annotation |
| --- | --- | --- | --- | --- | --- | --- | --- | --- | --- |
| 1 | 1 | 27139573 | | 28276669 | -0.9535 | 1137096 |  |  |  |
| 2 | 1 | 41881500 | | 42683552 | -0.8269 | 802052 |  |  |  |
| 3 | 2 | 3284455 | | 4120012 | -0.9446 | 835557 |  |  |  |
| 4 | 2 | 18294417 | | 18995957 | -0.781 | 701540 |  |  |  |
| 5 | 3 | 10476729 | | 12127559 | -0.8417 | 1650830 | 11000024 | 11499949 |  |
| 6 | 3 | 17981710 | | 22357563 | -0.803 | 4375853 | 15500099 | 15999986 |  |
| 6 | 3 | 17981710 | | 22357563 | -0.803 | 4375853 | 16000041 | 16499924 | TT1 |
| 7 | 4 | 16954242 | | 18024330 | -0.8624 | 1070088 |  |  |  |
| 8 | 4 | 22180925 | | 23452039 | -0.8852 | 1271114 |  |  |  |
| 9 | 4 | 26067433 | | 27238034 | -0.9473 | 1170601 |  |  |  |
| 10 | 4 | 33304379 | | 34877032 | -0.8152 | 1572653 |  |  |  |
| 11 | 5 | 13302265 | | 14204830 | -0.8846 | 902565 |  |  |  |
| 12 | 5 | 15413041 | | 16985694 | -0.7996 | 1572653 |  |  |  |
| 13 | 5 | 27910174 | | 28879748 | -0.8624 | 969574 | 28000036 | 28499964 |  |
| 14 | 6 | 4189074 | | 4857109 | -0.8496 | 668035 |  |  |  |
| 15 | 7 | 26022761 | | 26869486 | -0.7819 | 846725 |  |  |  |
| 16 | 7 | 26469486 | | 27293874 | -0.778 | 824388 | 28500040 | 28999964 | GLW7 |
| 17 | 9 | 805131 | | 1450830 | -0.808 | 645699 |  |  |  |
| 18 | 9 | 14039361 | | 14757653 | -0.8357 | 718292 |  |  |  |
| 19 | 10 | 18629461 | | 21207246 | -0.8441 | 2577785 | 20000047 | 20499919 |  |
| 20 | 12 | 10789436 | | 11457471 | -0.7697 | 668035 |  |  |  |
| 21 | 12 | 12576336 | | 13936795 | -0.8685 | 1360459 | 15500049 | 15999987 |  |
| 22 | 12 | 24682585 | | 25350620 | -0.8424 | 668035 |  |  |  |

**Supplementary Table 5** Table for the genome coordinate of the 13 consistently high genetic introgression regions between the three subgroups of rice population

| Serial | Chro | Block | Start | End |
| --- | --- | --- | --- | --- |
| 1 | 2 | 40 | 19500009 | 19999980 |
| 2 | 4 | 32 | 15500023 | 15999999 |
| 3 | 4 | 48 | 23500013 | 23999991 |
| 4 | 5 | 19 | 9000052 | 9499981 |
| 5 | 5 | 24 | 11500028 | 11999939 |
| 6 | 5 | 25 | 12000159 | 12499985 |
| 7 | 5 | 26 | 12500206 | 12999988 |
| 8 | 5 | 27 | 13000007 | 13499951 |
| 9 | 5 | 31 | 15000008 | 15499984 |
| 10 | 5 | 32 | 15500022 | 15999863 |
| 11 | 6 | 8 | 3501660 | 3999889 |
| 12 | 6 | 13 | 6000003 | 6499983 |
| 13 | 7 | 58 | 28500040 | 28999964 |

**Supplementary Table 6** Table for the introgression ratio within the rice subgroups and their progenitors in 13 consistently high genetic introgression regions

| Serial | Indica_in_temp | Temp_in_indica | Trop_in_indica | Trop_in_Or-III | Temp_in_Or-III | Indica_in_Or-I | Indica_in_trop |
| --- | --- | --- | --- | --- | --- | --- | --- |
| 1 | 8.46% | 13.45% | 45.33% | 0.00% | 1.47% | 1.92% | 4.04% |
| 2 | 9.81% | 19.56% | 33.33% | 0.00% | 4.89% | 13.08% | 10.58% |
| 3 | 9.62% | 16.38% | 36.00% | 0.00% | 1.71% | 8.65% | 7.69% |
| 4 | 16.92% | 17.60% | 32.00% | 0.00% | 1.71% | 3.65% | 9.23% |
| 5 | 9.42% | 23.23% | 44.00% | 5.33% | 4.89% | 10.00% | 11.92% |
| 6 | 7.88% | 24.69% | 33.33% | 4.00% | 6.11% | 11.54% | 14.62% |
| 7 | 8.85% | 27.38% | 40.00% | 2.67% | 4.16% | 10.00% | 15.77% |
| 8 | 14.23% | 38.39% | 58.67% | 0.00% | 2.93% | 7.88% | 21.73% |
| 9 | 10.19% | 13.45% | 53.33% | 0.00% | 0.98% | 0.96% | 2.88% |
| 10 | 9.04% | 14.18% | 57.33% | 8.00% | 9.29% | 8.85% | 2.88% |
| 11 | 9.81% | 15.89% | 37.33% | 0.00% | 0.49% | 4.04% | 7.12% |
| 12 | 17.69% | 14.43% | 68.00% | 14.67% | 1.22% | 3.85% | 1.54% |
| 13 | 14.81% | 17.85% | 86.67% | 1.33% | 1.96% | 2.12% | 1.54% |


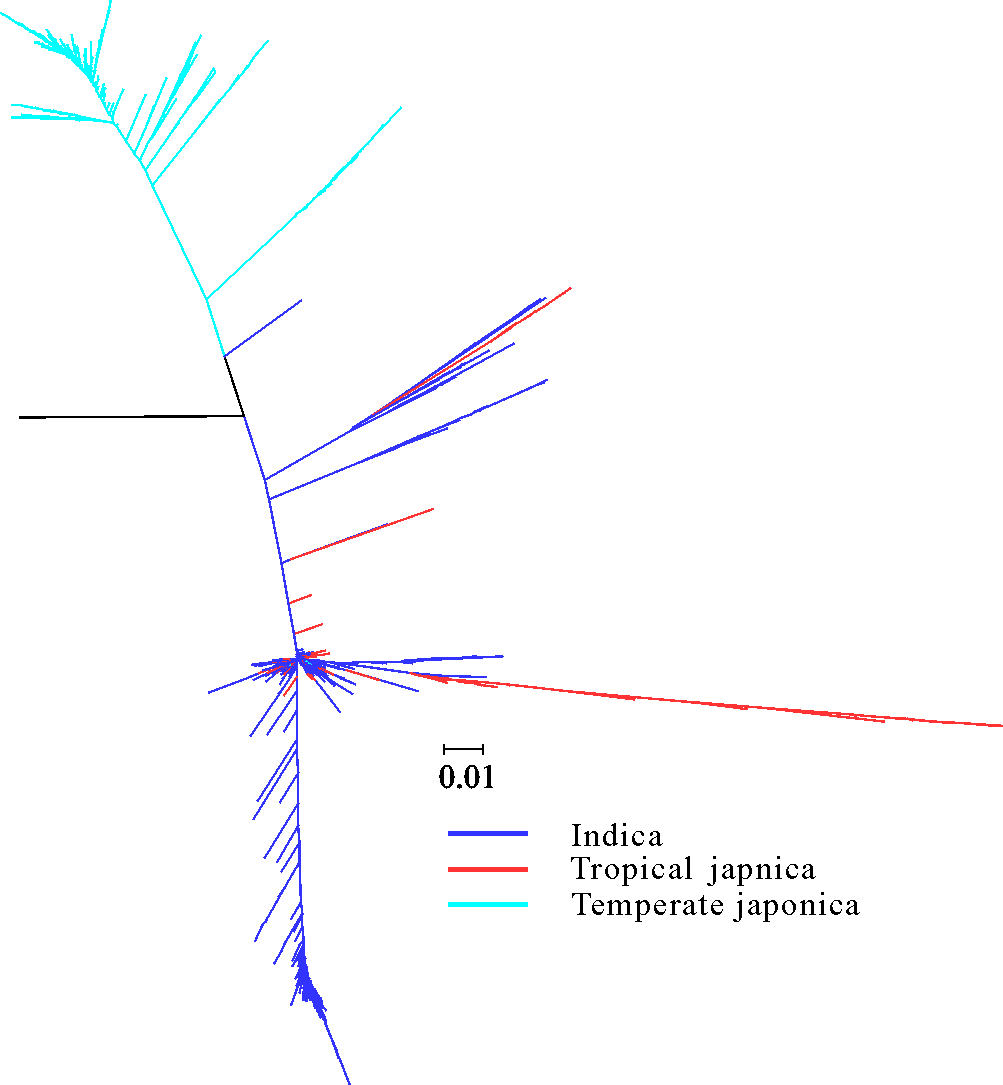


**Supplementary Figure1**. Neighbor-joining tree constructed with the 500kb flanking genetic variants around the *TT1* locus. The African cultivated rice is used as the outgroup. The accessions of different groups are labeled as illustrated in the Figure.


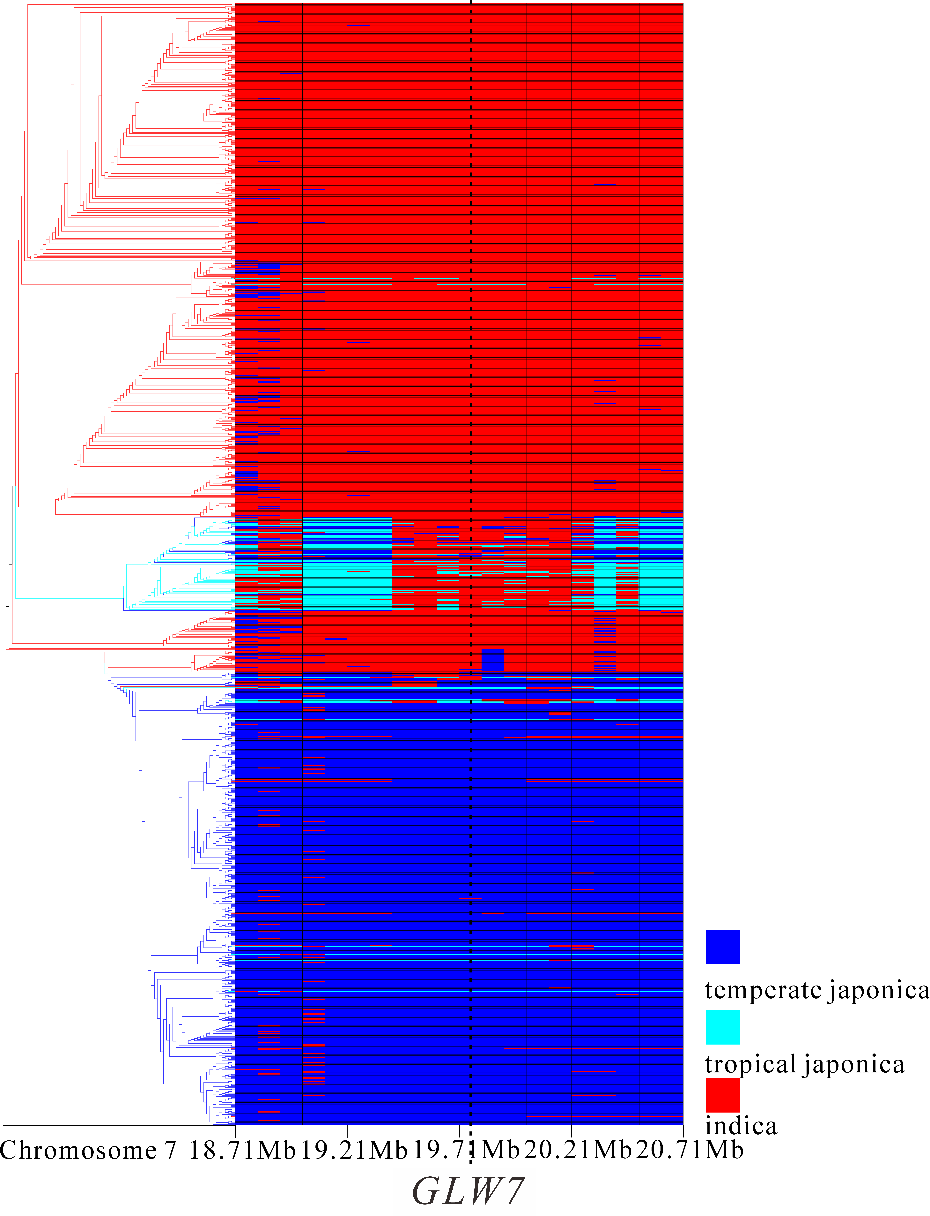


**Supplementary Figure2**. Diagram for the haplotype around the 1Mb flanking genetic variant around the GLW7 locus. The horizontal line represents the 2Mb genetic region around the GLW7 locus. They are divided into 100Kb small blocks. The Vertical line represents the whole rice population. Each horizontal line represents one accession. The dotted line represents the position for the GLW7. The phylogenetic tree on the left represents the relationship of the whole population. It’s constructed with the genetic variants located in the 2Mb around the GLW7. The rice accessions are colored, as illustrated in the diagram.


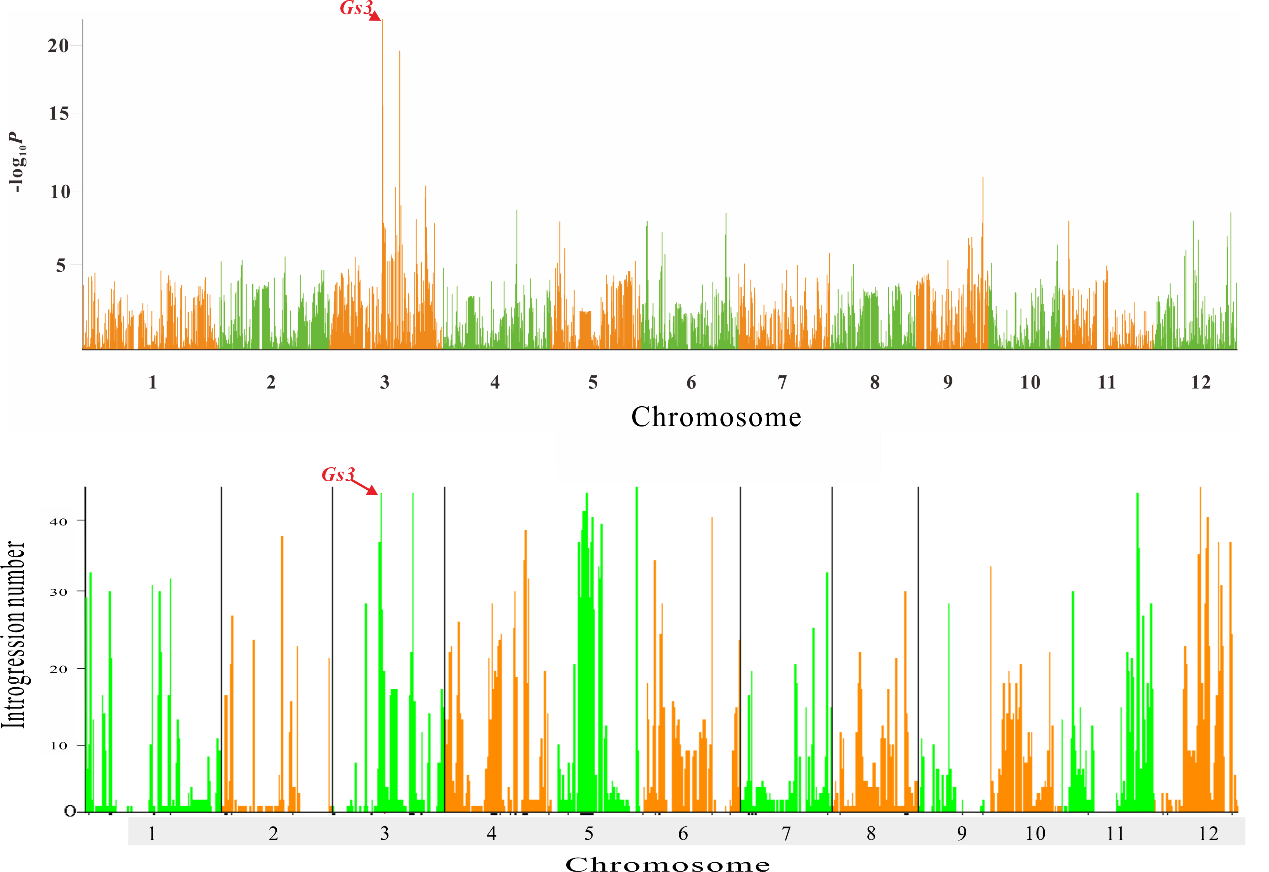


**Supplementary Figure3**. Genetic introgression in the GS3 locus between the *indica* subgroup and the japonica group. Figure A represents the admixture association mapping result for the grain length phenotype using the *indica* component matrix. The horizontal line represents the 12 chromosomes of the rice genome. The vertical line represents the transformed association P-value. Figure B represents the *indica* introgression accession number detected between the *indica* and the *tropical japonica* subgroups. The vertical line is the *indica* introgression accession number.


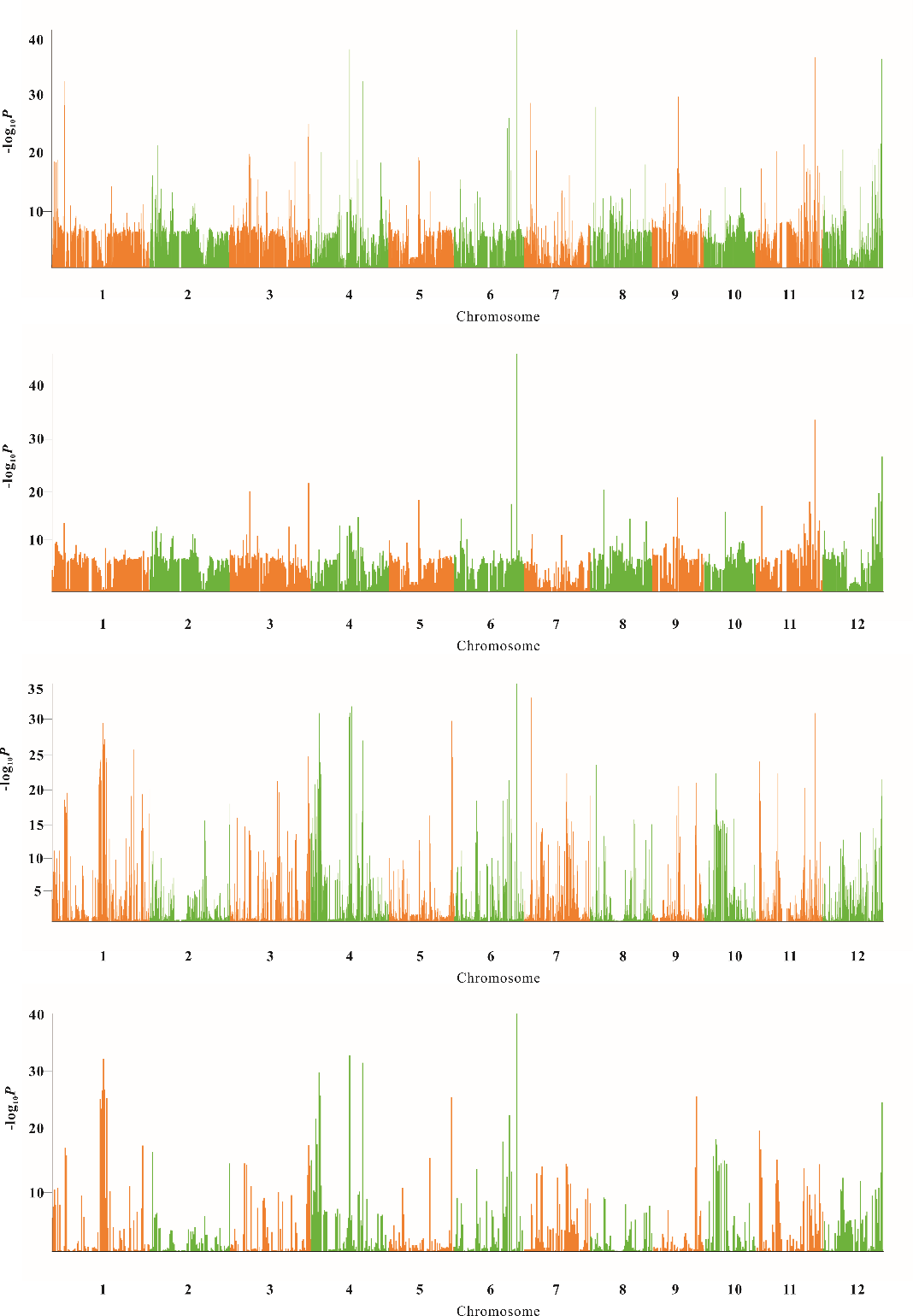


**Supplementary Figure4**. Admixture association mapping results using heading date phenotype. From the top to down, figure 1 shows the association result of the *indica* component detected in the merged population of *indica* and temperate population. Figure 2 shows the association result of the *temperate japonica* component detected in the merged population of *temperate japonica* and *indica*. Figure 3 shows the association result of the *tropical japonica* component detected in the merged population of *tropical japonica* and *indica*. Figure 4 shows the association result of the *indica* component detected in the merged population of *tropical japonica* and *indica*.


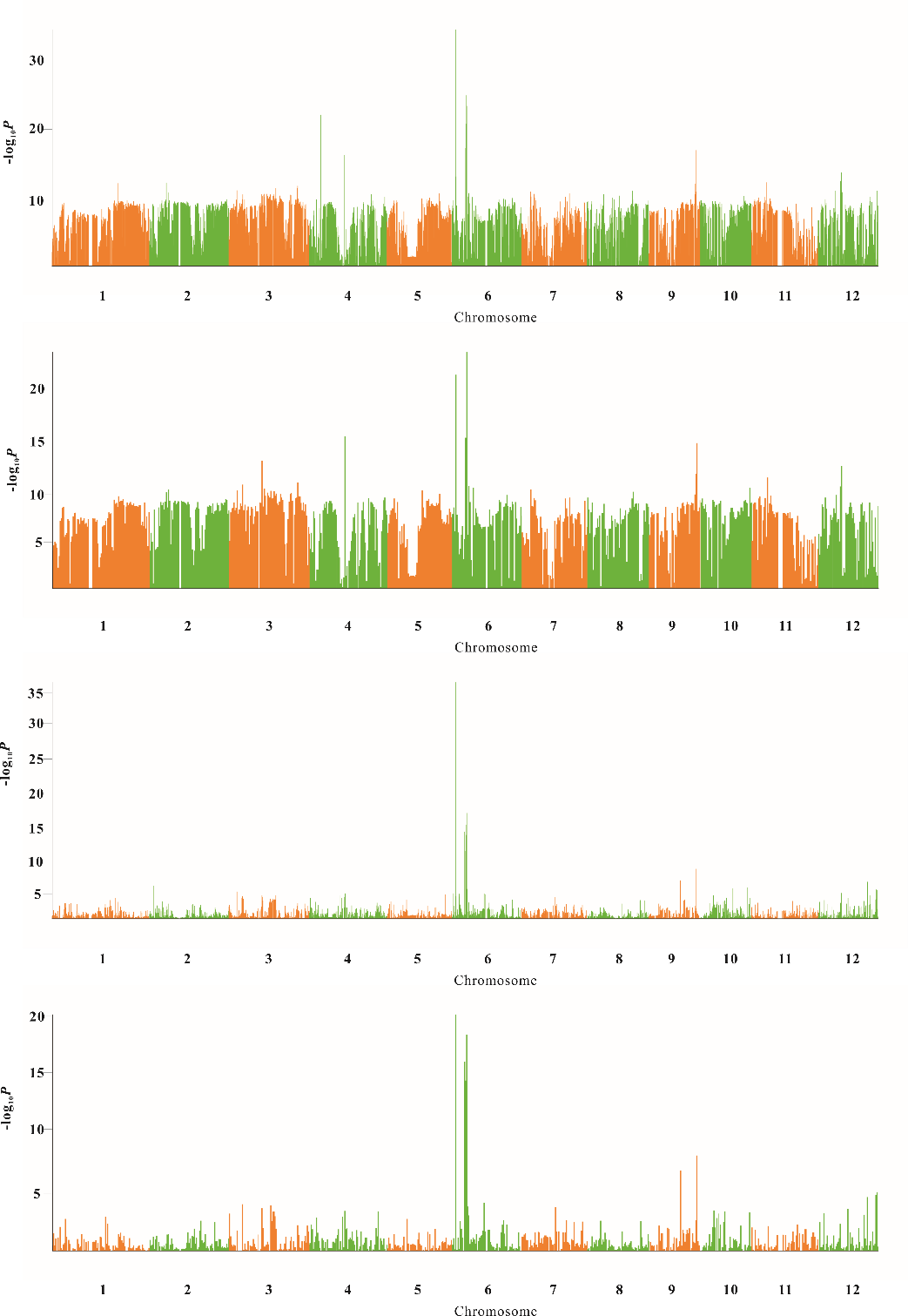


**Supplementary Figure5**. Admixture association mapping results using amylose content phenotype. From the top to down, figure 1 shows the association result of the *indica* component detected in the merged population of *indica* and temperate population. Figure 2 shows the association result of the *temperate japonica* component detected in the merged population of *temperate japonica* and *indica*. Figure 3 shows the association result of the *tropical japonica* component detected in the merged population of *tropical japonica* and *indica*. Figure 4 shows the association result of the *indica* component detected in the merged population of *tropical japonica* and *indica*.


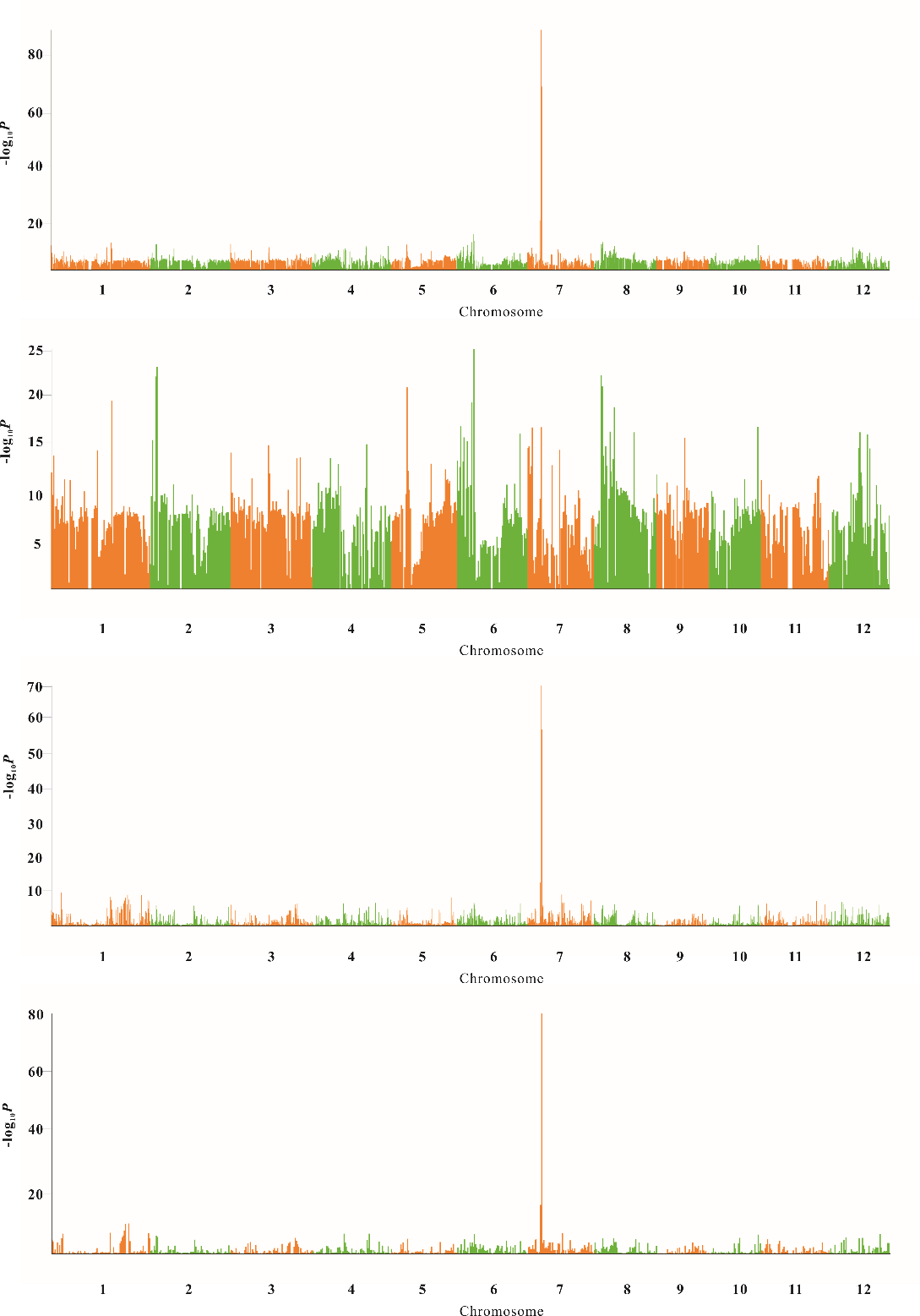


**Supplementary Figure6**. Admixture association mapping results using pericarp color phenotype. From the top to down, figure 1 shows the association result of the *indica* component detected in the merged population of *indica* and temperate population. Figure 2 shows the association result of the *temperate japonica* component detected in the merged population of *temperate japonica* and *indica*. Figure 3 shows the association result of the *tropical japonica* component detected in the merged population of *tropical japonica* and *indica*. Figure 4 shows the association result of the *indica* component detected in the merged population of *tropical japonica* and *indica*.


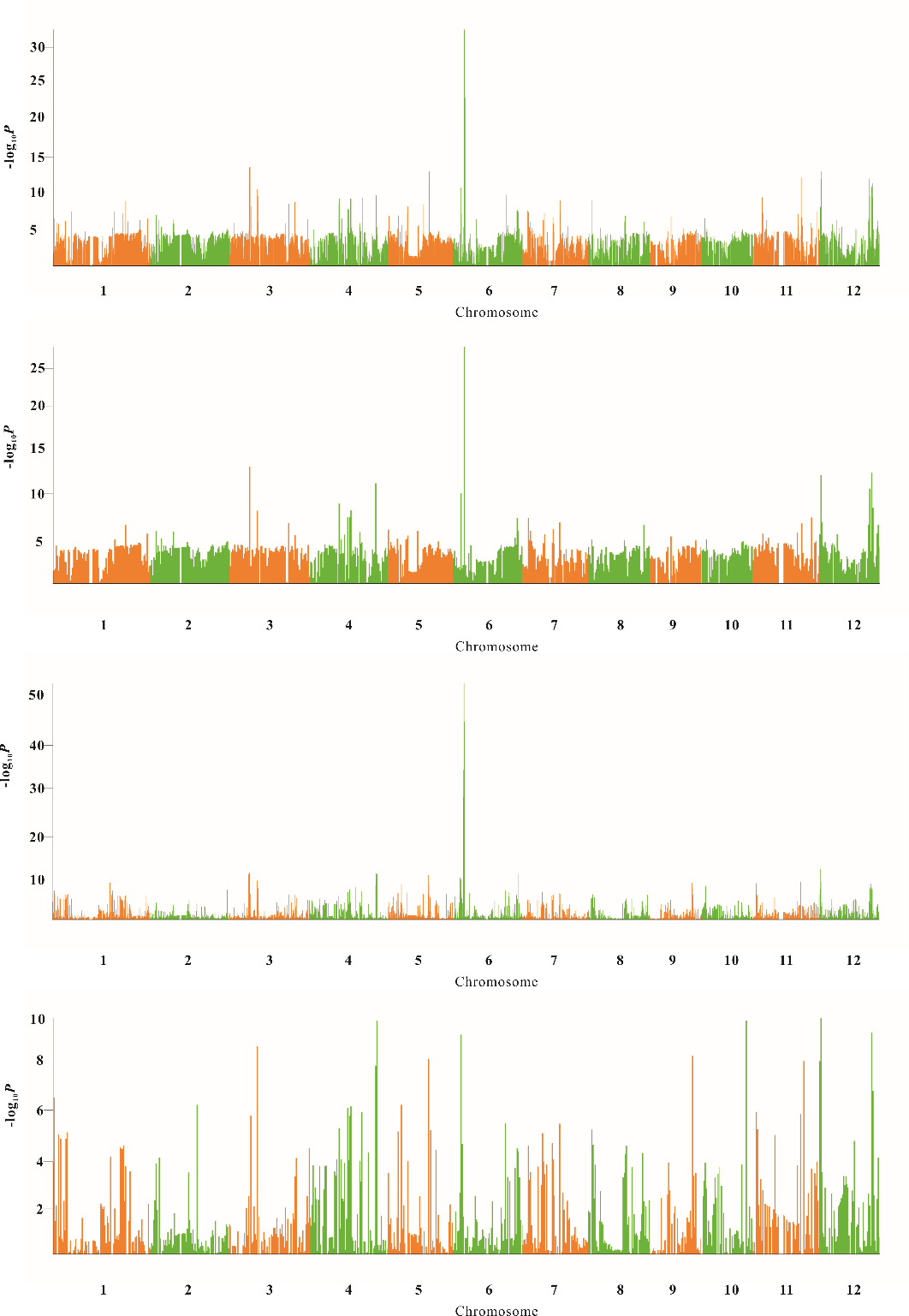


**Supplementary Figure7**. Admixture association mapping results using tip color phenotype. From the top to down, figure 1 shows the association result of the *indica* component detected in the merged population of *indica* and temperate population. Figure 2 shows the association result of the *temperate japonica* component detected in the merged population of *temperate japonica* and *indica*. Figure 3 shows the association result of the *tropical japonica* component detected in the merged population of *tropical japonica* and *indica*. Figure 4 shows the association result of the *indica* component detected in the merged population of *tropical japonica* and *indica*.


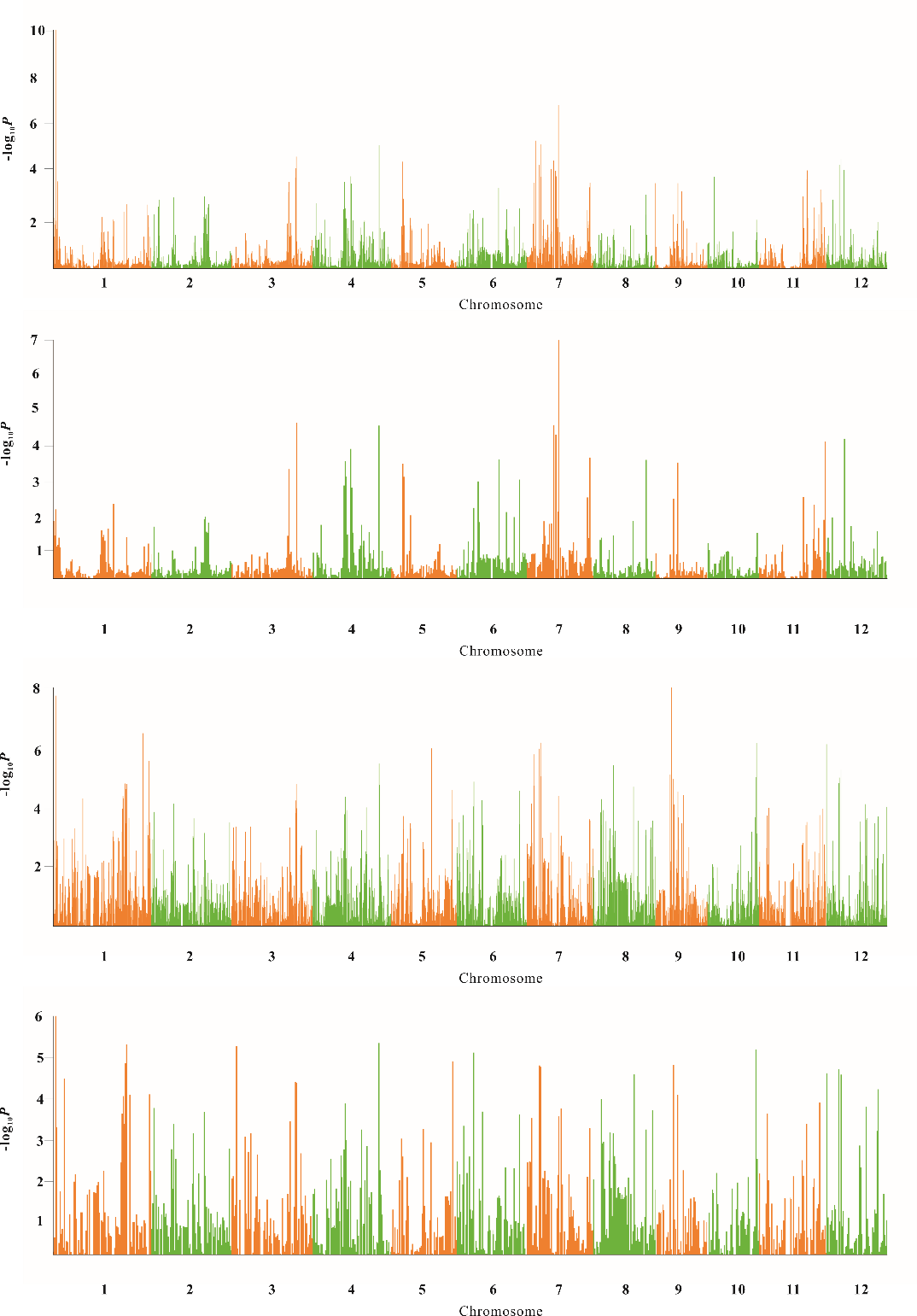


**Supplementary Figure8**. Admixture association mapping results using glue color phenotype. From the top to down, figure 1 shows the association result of the *indica* component detected in the merged population of *indica* and temperate population. Figure 2 shows the association result of the *temperate japonica* component detected in the merged population of *temperate japonica* and *indica*. Figure 3 shows the association result of the *tropical japonica* component detected in the merged population of *tropical japonica* and *indica*. Figure 4 shows the association result of the *indica* component detected in the merged population of *tropical japonica* and *indica*.


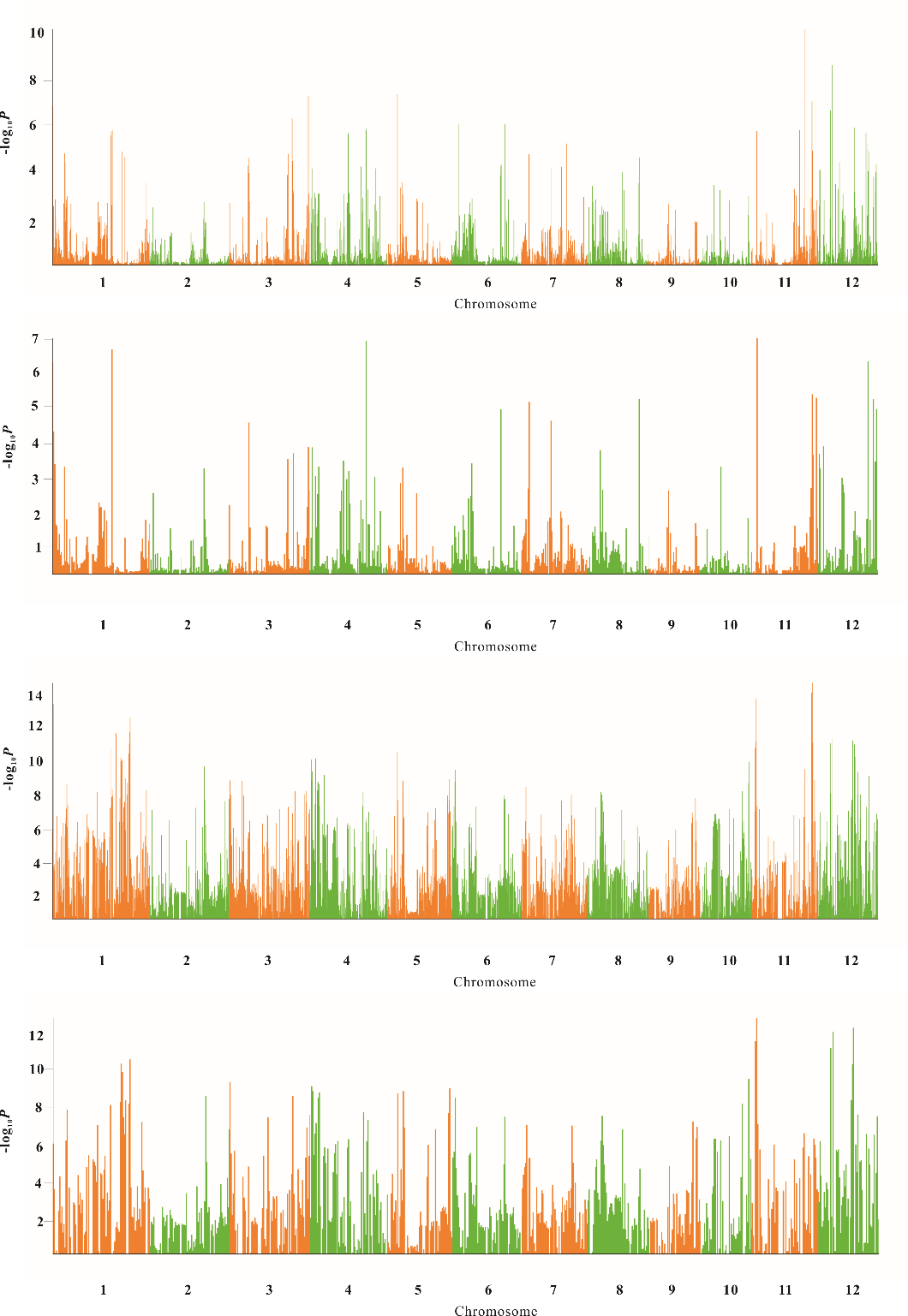


**Supplementary Figure9**. Admixture association mapping results using protein content phenotype. From the top to down, figure 1 shows the association result of the *indica* component detected in the merged population of *indica* and temperate population. Figure 2 shows the association result of the *temperate japonica* component detected in the merged population of *temperate japonica* and *indica*. Figure 3 shows the association result of the *tropical japonica* component detected in the merged population of *tropical japonica* and *indica*. Figure 4 shows the association result of the *indica* component detected in the merged population of *tropical japonica* and *indica*.


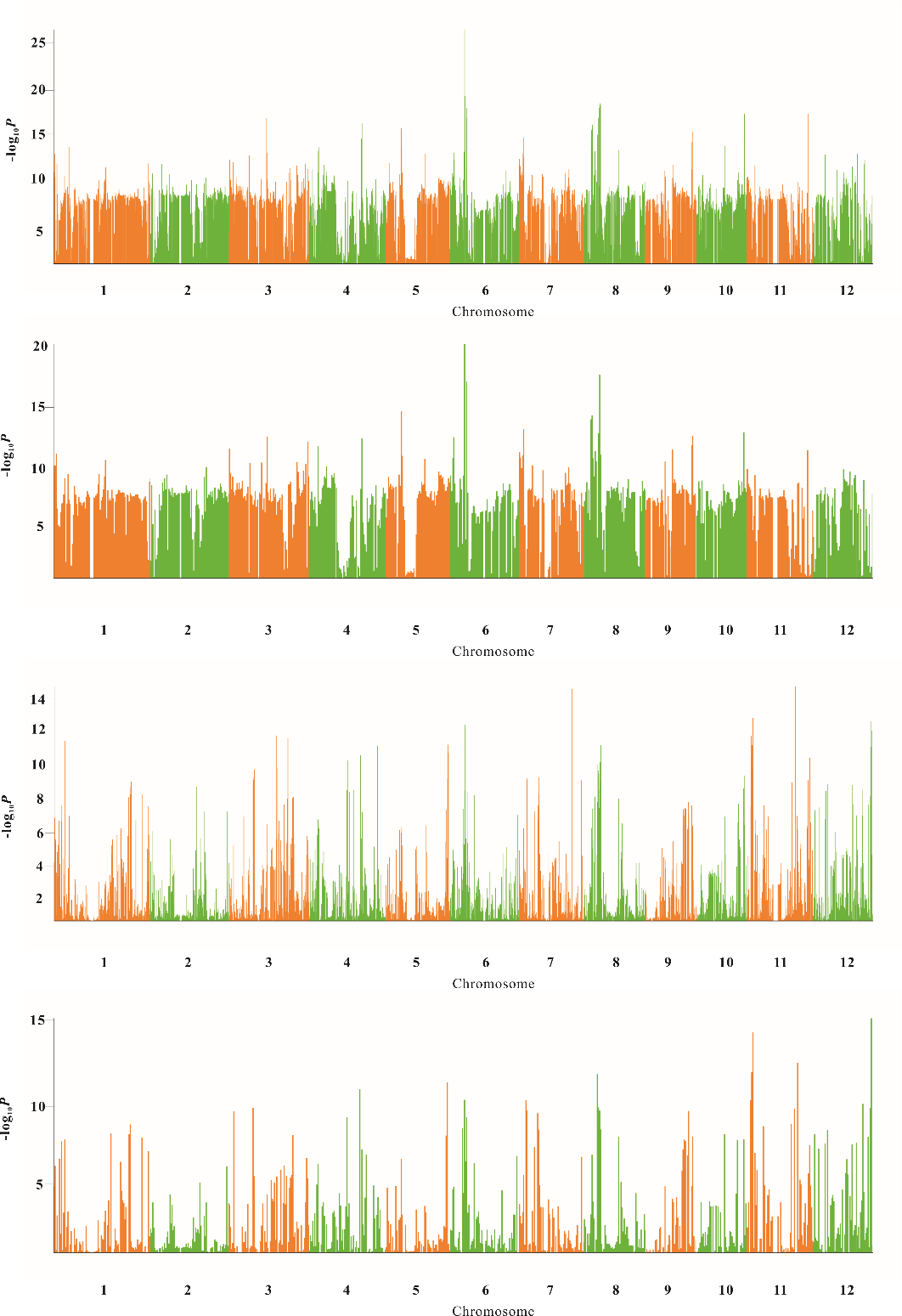


**Supplementary Figure10**. Admixture association mapping results using gelatinization temperature phenotype. From the top to down, figure 1 shows the association result of the *indica* component detected in the merged population of *indica* and temperate population. Figure 2 shows the association result of the *temperate japonica* component detected in the merged population of *temperate japonica* and *indica*. Figure 3 shows the association result of the *tropical japonica* component detected in the merged population of *tropical japonica* and *indica*. Figure 4 shows the association result of the *indica* component detected in the merged population of *tropical japonica* and *indica*.


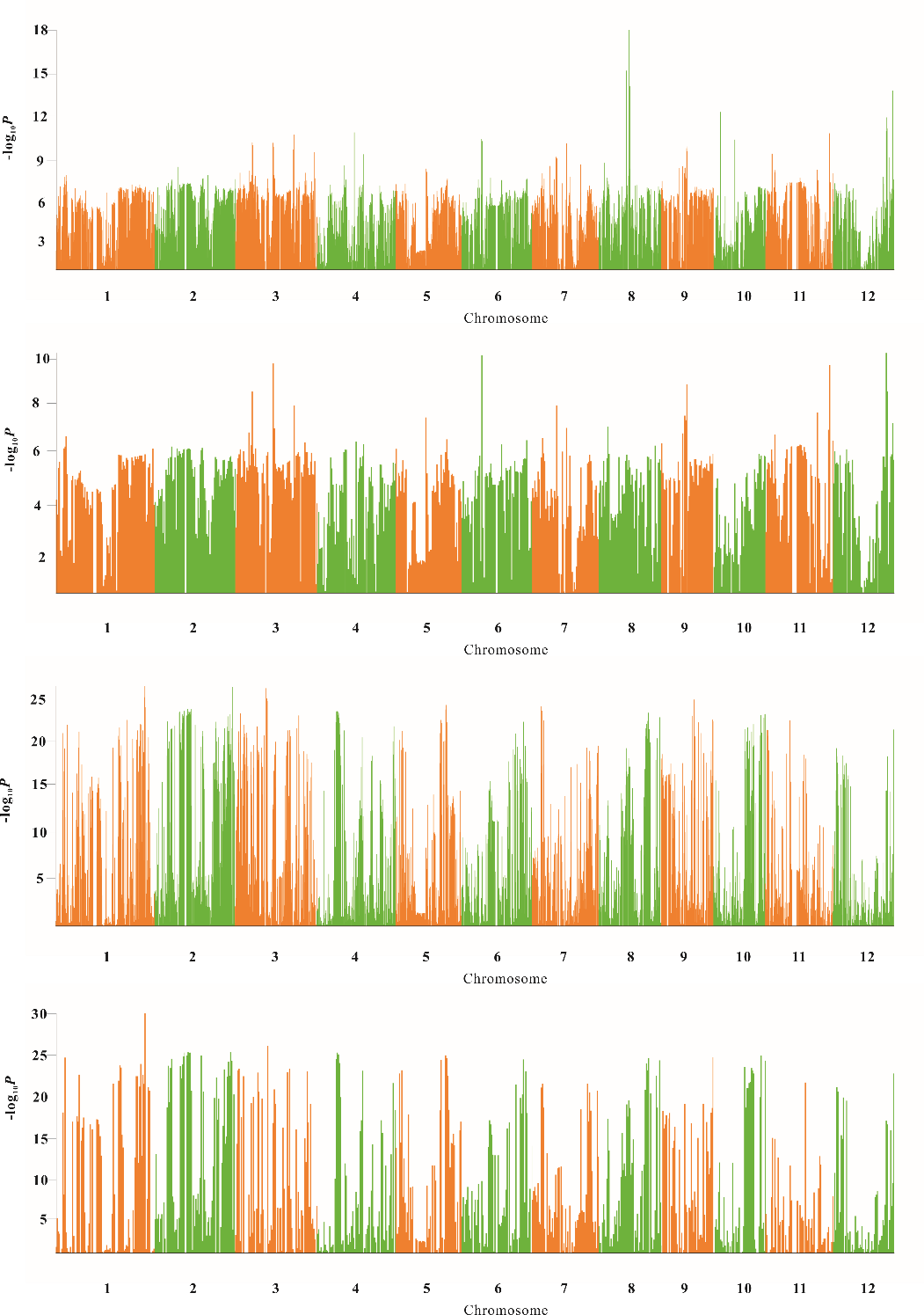


**Supplementary Figure11**. Admixture association mapping results using thousand-grain weight(TGW) phenotype. From the top to down, figure 1 shows the association result of the *indica* component detected in the merged population of *indica* and temperate population. Figure 2 shows the association result of the temperature japonica component detected in the merged population of *temperate japonica* and *indica*. Figure 3 shows the association result of the *tropical japonica* component detected in the merged population of *tropical japonica* and *indica*. Figure 4 shows the association result of the *indica* component detected in the merged population of *tropical japonica* and *indica*.


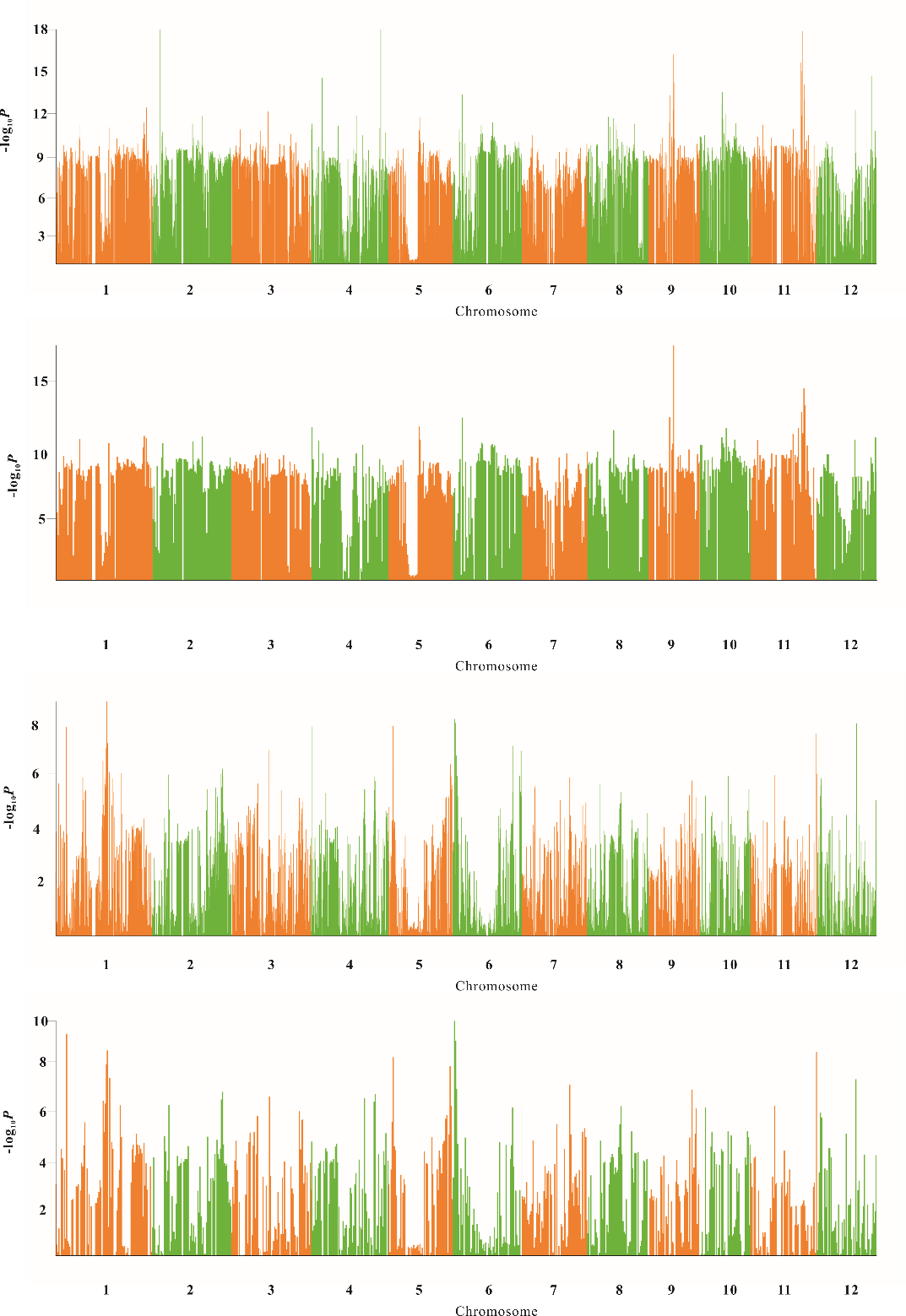


**Supplementary Figure12**. Admixture association mapping results using grain number per panicle phenotype. From the top to down, figure 1 shows the association result of the *indica* component detected in the merged population of *indica* and temperate population. Figure 2 shows the association result of the *temperate japonica* component detected in the merged population of *temperate japonica* and *indica*. Figure 3 shows the association result of the *tropical japonica* component detected in the merged population of *tropical japonica* and *indica*. Figure 4 shows the association result of the *indica* component detected in the merged population of *tropical japonica* and *indica*.


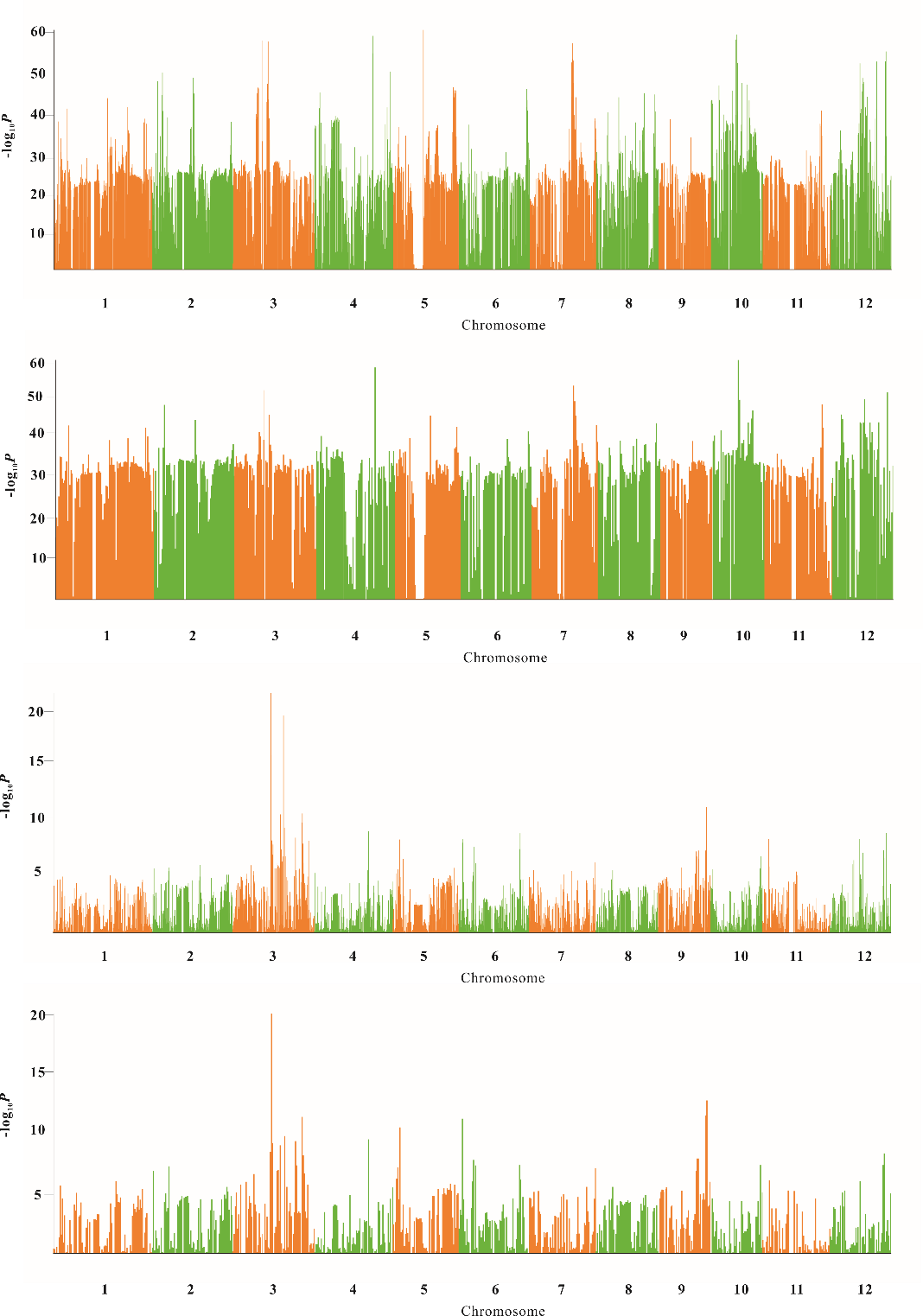


**Supplementary Figure13**. Admixture association mapping results using grain length phenotype. From the top to down, figure 1 shows the association result of the *indica* component detected in the merged population of *indica* and temperate population. Figure 2 shows the association result of the *temperate japonica* component detected in the merged population of *temperate japonica* and *indica*. Figure 3 shows the association result of the *tropical japonica* component detected in the merged population of *tropical japonica* and *indica*. Figure 4 shows the association result of the *indica* component detected in the merged population of *tropical japonica* and *indica*.


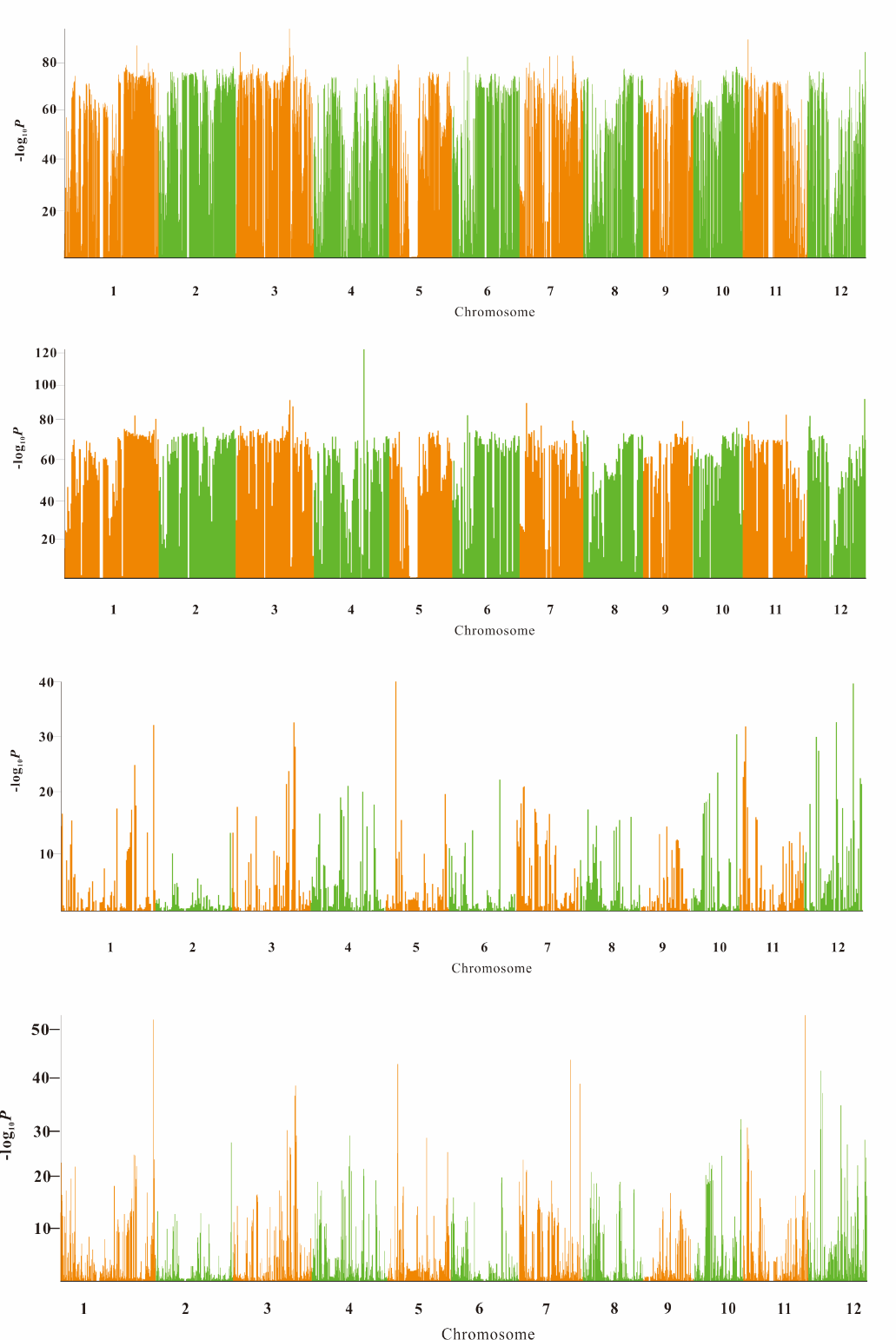


**Supplementary Figure14**. Admixture association mapping results using grain width phenotype. From the top to down, figure 1 shows the association result of the *indica* component detected in the merged population of *indica* and temperate population. Figure 2 shows the association result of the *temperate japonica* component detected in the merged population of *temperate japonica* and *indica*. Figure 3 shows the association result of the *tropical japonica* component detected in the merged population of *tropical japonica* and *indica*. Figure 4 shows the association result of the *indica* component detected in the merged population of *tropical japonica* and *indica*.


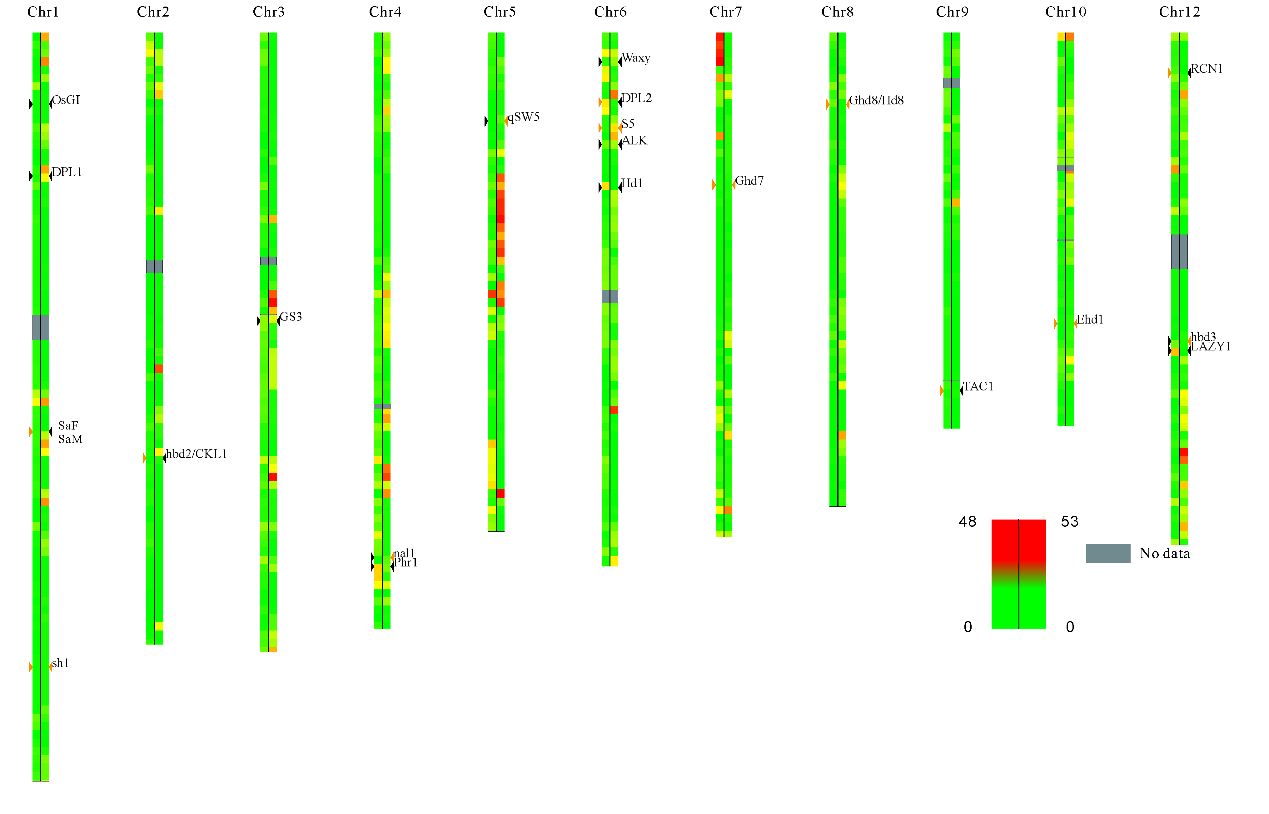


**Supplementary Figure15**. The introgression between the *indica* and the *tropical japonica* overlaps with the rice known agronomically important genes. The left side of each chromosome represents the genetic introgression number from the *tropical japonica* to the *indica* population. The right side of each chromosome represents the genetic introgression from the *tropical japonica* to the *indica* population. The color change from green to red indicates higher introgression accessions.


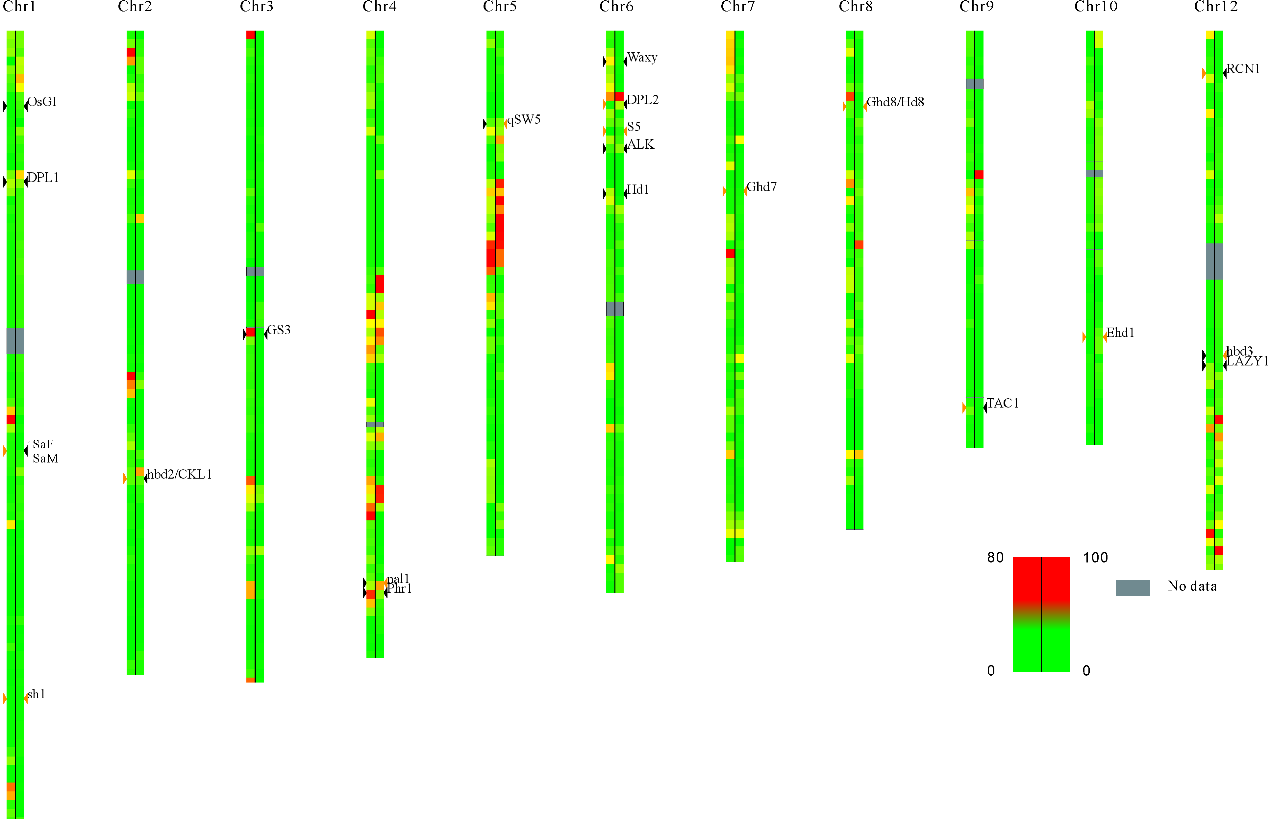


**Supplementary Figure16**. The introgression between the *indica* and the temperature japonica overlapping with the rice known agronomically important genes. The left side of each chromosome represents the genetic introgression number from the temperature japonica to the *indica* population. The right side of each chromosome represents the genetic introgression from the temperature japonica to the *indica* population. The color change from green to red indicates higher introgression accessions.


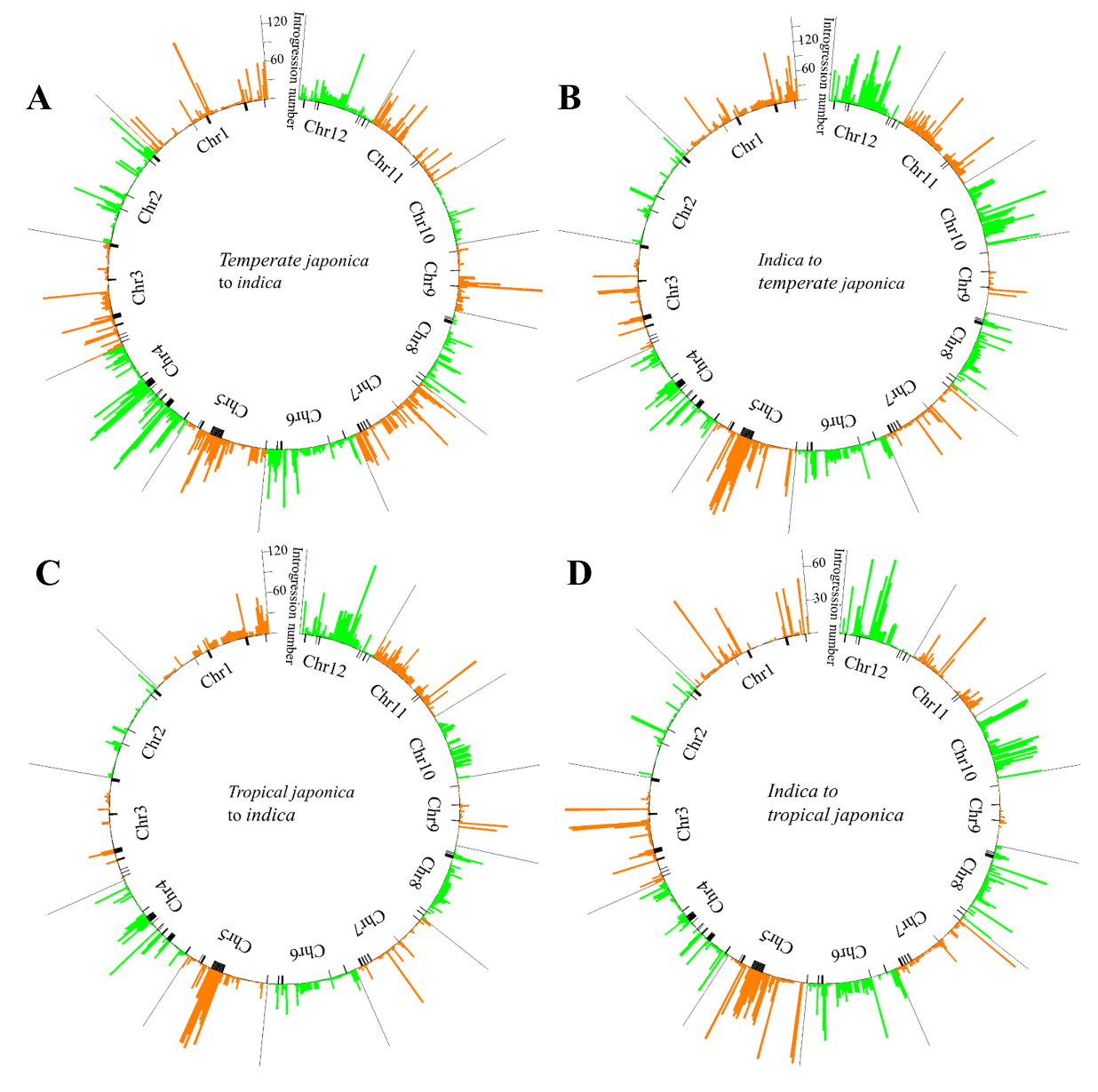


**Supplementary Figure17**. Summary of the cultivated rice accessions that exist introgression between different groups in circles. The vertical line on the outside the circle represents the number of accessions with genetic introgression between different groups. The black line inside the circle represents the 55 major selective sweeps detected by the previous study. Figure A represents summary data for genetic introgression from the *temperate japonica* to the *indica* subgroup. Figure B represents the summary data for the genetic introgression from the *indica* to the *temperate japonica*. Figure C represents the summary data for the genetic introgression from the *tropical japonica* to the *indica*. Figure D represents the summary data for the genetic introgression from the *indica* to the *tropical japonica*.


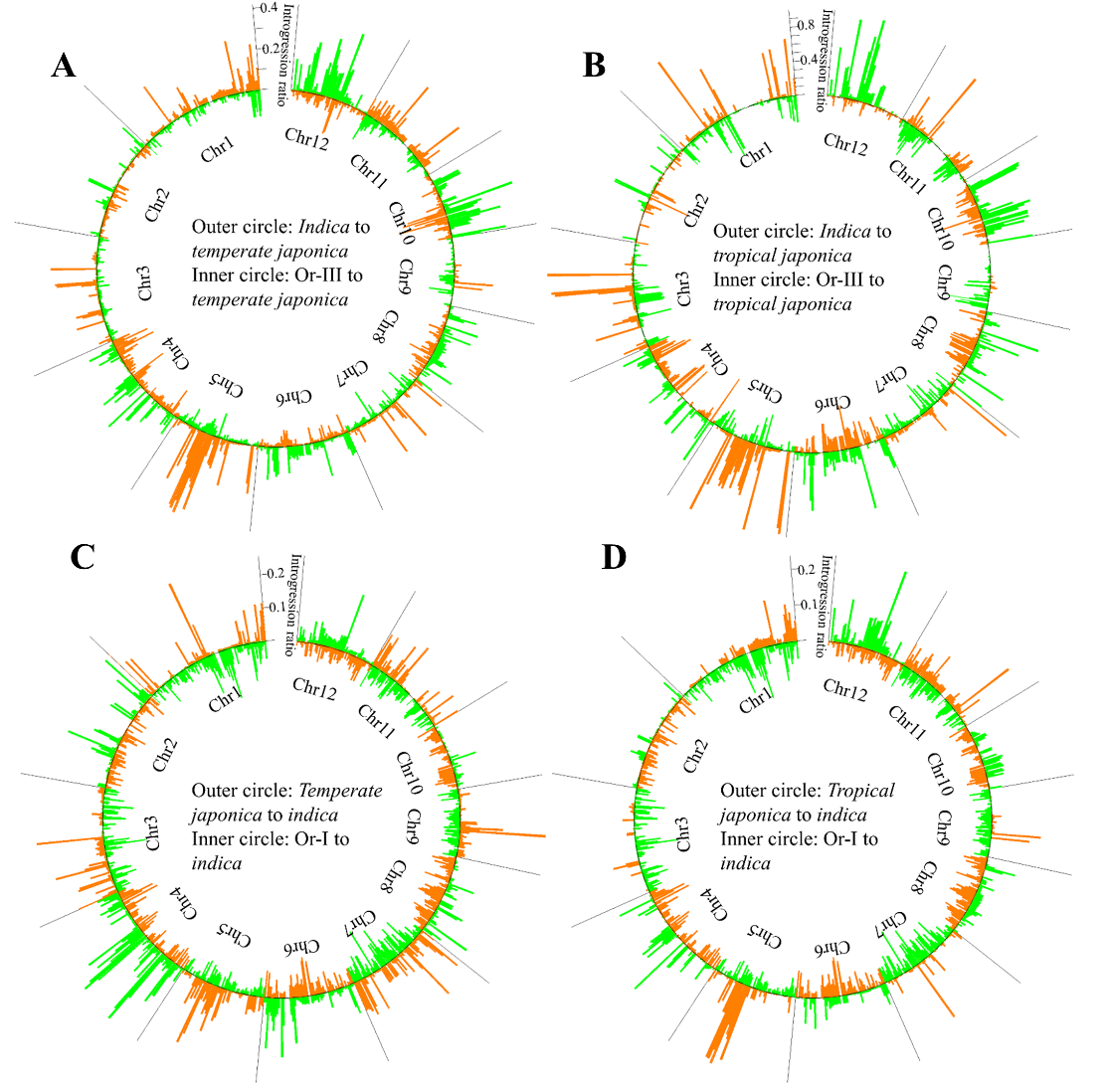


**Supplementary Figure18.** Summary of the genetic introgression between the rice subgroup and their wild rice progenitors. Figure A shows the genetic introgression from *indica* to *temperate japonica* in the outer circle, the inner circle shows the introgression from Or-III to *temperate japonica*. Figure B shows the genetic introgression from *indica* to *tropical japonica* in the outer circle, the inner circle shows the introgression from Or-III to *tropical japonica*. Figure C shows the genetic introgression from *temperate japonica* to *indica* in the outer circle, the inner circle shows the introgression from Or-I to *indica*. Figure D shows the genetic introgression from *tropical japonica* to *indica* in the outer circle, the inner circle shows the introgression from Or-I to *indica*.
